# Distilling direct effects via conditional differential gene expression analysis

**DOI:** 10.1093/bioinformatics/btag784

**Published:** 2026-10-20

**Authors:** Jiaqi Gu, Andrew Skelton, James Staley, Pierce Popson, Lei Peng, Xiaoyu Song, Juliet Knowles, Zihuai He

**Affiliations:** Department of Mathematics and Statistics, University of South Florida, Tampa, FL 33620, United States; UCB Pharma, Slough, SL1 3WE, United Kingdom; UCB Pharma, Slough, SL1 3WE, United Kingdom; Department of Neurology and Neurological Sciences, Stanford University, Stanford, CA 94305, United States; Department of Neurology and Neurological Sciences, Stanford University, Stanford, CA 94305, United States; Centre for Quantitative Medicine, Duke-NUS Medical School, 169857, Singapore; Department of Neurology and Neurological Sciences, Stanford University, Stanford, CA 94305, United States; Department of Neurology and Neurological Sciences, Stanford University, Stanford, CA 94305, United States; Quantitative Sciences Unit, Department of Medicine, Stanford University, Palo Alto, CA 94305, United States; Department of Biomedical Data Science, Stanford University, Stanford, CA 94305, United States

## Abstract

**Motivation:**

Differential gene expression (DGE) analysis is foundational for interpreting RNA sequencing data, but it conflates direct biological effects with correlations propagated through gene co-expression. Across three RNA sequencing datasets (including a genome-scale perturb-seq experiment), we find that only a small fraction of differentially expressed genes have direct effects on the trait of interest, while the majority are undirected or passengers whose associations are mediated through other genes.

**Results:**

To distinguish direct effect genes, we introduce conditional differential gene expression (CDGE) analysis, a framework that tests for conditional rather than marginal association between each gene and the trait of interest. Implemented via the GhostKnockoff procedure with lasso regression, CDGE delivers false discovery rate control, operates on summary statistics from existing DGE pipelines, and accommodates batch effects. The genes identified by CDGE mediate the effects of most other differentially expressed genes and show stronger enrichment for known protein–protein interactions and biological pathways than DGE-identified genes. These results suggest that the field has been systematically over-interpreting DGE outputs, and that distinguishing direct from mediated effects is essential for prioritizing genes for functional follow-up and therapeutic development.

**Availability and implementation:**

To facilitate public access to knockoff-based CDGE analysis, we have developed the R package “KnockoffCDGE” as an integrated implementation, which is freely available at https://github.com/GuJQ5/KnockoffCDGE (DOI: 10.5281/zenodo.23241517).

## 1 Introduction

The single-cell RNA sequencing (scRNA-seq) technology has provided extensive data on diseases and biological processes (Jovic *et al.* 2022, Nesari *et al.* 2026). As the state-of-the-art approach, scRNA-seq is used to measure transcriptome expression levels in individual cells, enabling the study of cellular processes and biological pathways. Correspondingly, multiple statistical methods have been proposed to analyze these data, among which differential gene expression (DGE) analysis is one popular category (Costa-Silva *et al.* 2017, Kebschull *et al.* 2017, Jones *et al.* 2024, Rosati *et al.* 2024). DGE analysis identifies genes associated with traits of interest (e.g. diseases or gene perturbations) by comparing gene expression levels between cells of different conditions (e.g. healthy versus disease cells/tissues, control cells versus perturbed cells) under either parametric model (Ho *et al.* 2008, Anders and Huber 2010, Hardcastle and Kelly 2010, Robinson *et al.* 2010, Wang *et al.* 2010, Leng *et al.* 2013, Love *et al.* 2014, Ritchie *et al.* 2015, Ran and Daye 2017) or nonparametric model (Tarazona *et al.* 2012, 2015; Li and Tibshirani 2013). These technologies have offered unprecedented opportunities to investigate and understand the underlying biological mechanisms of diseases or patterns of gene–gene interactions, especially with the aid of external information from functional enrichment analysis (Udhaya Kumar *et al.* 2020, Rosati *et al.* 2024).

Although widely utilized, most DGE analyses encounter difficulties in distinguishing between genes on the biological pathways and those not. This limitation arises because differentially expressed genes are identified based on their expression levels being marginally associated with the trait of interest, while the potential confounding brought by gene coexpression is ignored (Farahbod and Pavlidis 2019). Instead of acting alone, genes and their corresponding proteins coordinate as functional modules or aggregate to form nano-machineries in biological processes (Lui *et al.* 2015). Thus, genes in same biological processes have similar expression patterns and strongly correlated expression levels (Boutros and Okey 2005, Do and Choi 2008). Without accounting for expression level correlations induced by functional interdependence, it is likely that genes not on the biological pathways are also identified as differentially expressed. For example, in Fig. 1a where we visualize gene-disease relations, there are 4 types of genes as follows.

**Figure 1:**
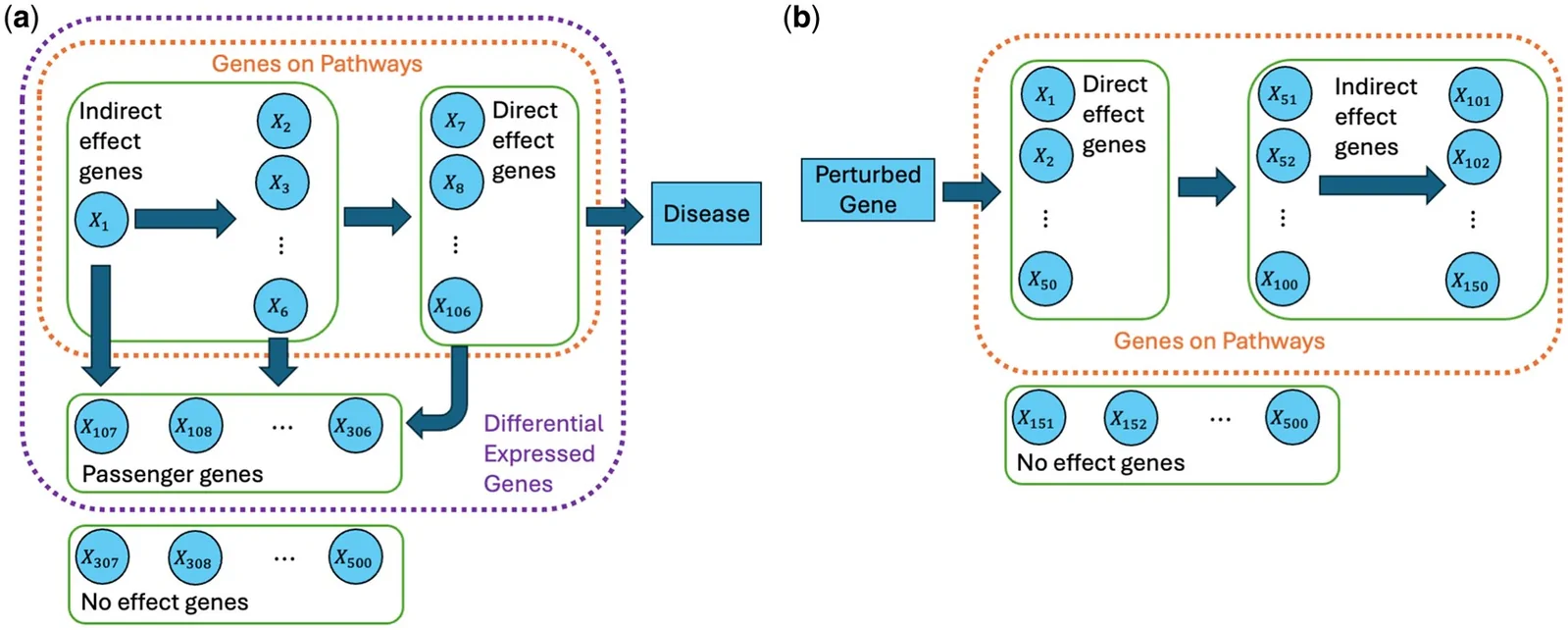
Graphical illustration of different types of genes when (a) investigating gene-disease relations or (b) investigating gene–gene relations.

- **Direct effect genes** are involved in the pathways of the disease and are the closest precedents of the disease (genes $X_{7},X_{8},\ldots ,X_{106}$).
- **Indirect effect genes** are involved in the pathways of the disease and are not the closest precedents of the disease (genes $X_{1},X_{2},\ldots ,X_{6}$).
- **Passenger genes** are not involved in the pathways of the disease but are affected by indirect effect genes or direct effect genes (e.g. genes $X_{107},X_{108},\ldots ,X_{306}$).
- **No effect genes** are not involved in the pathways of the disease and are not affected by indirect effect genes or direct effect genes (genes $X_{307},X_{308},\ldots ,X_{500}$).

Expression levels of the first three types of genes are expected to correlate with the disease of interest. We use the term “passenger” here in deliberate analogy to its usage in cancer genomics, where passenger mutations are correlated with the tumor phenotype but not causally upstream of it (Stratton *et al.* 2009, Vogelstein *et al.* 2013); we extend the term from the mutation context to the expression context, with the same intuition that a passenger is correlated with the trait of interest but downstream of it or genes on the pathways. As a result, the DGE analysis would identify not only the genes on the pathways (first two types) but also passenger genes (the third type). These redundantly identified genes would obscure the most important target genes when designing biological studies for functional validation or developing medicines. Under particular conditions, direct effects of genes can be translated to causal effects, and identifying genes with direct effects helps reveal the “wiring” of biological circuits and benefit medicine development. For example, therapies targeting direct effect genes or their products improve efficacy (Jiang *et al.* 2019) and reduce toxicity (Xu *et al.* 2022), with consistent benefits across different populations (Slamon *et al.* 2001, 2011). In contrast, drugs targeting indirect effect genes or passenger genes show less consistent effects (Singh and Chakrabarti 2023, Arias-Badia *et al.* 2025) and higher risks of adverse events (Donath and Shoelson 2011). The need to identify direct effect genes also arises when we investigate gene expression level variations in genetic perturbation experiments (as visualized in Fig. 1b). Identifying direct effects from perturbed genes to measured genes can help to establish our knowledge of gene–gene relations and benefit medical practice. For example, in therapies that modify gene expression, we can monitor expression levels of only direct effect genes and truncate unwanted mutation effects with fewer drugs. This calls for statistical approaches that can pinpoint genes with direct effects.

In this article, we argue that DGE analysis identifies the right set of trait-relevant genes but cannot distinguish drivers from passengers within that set, leading to systematic misallocation of follow-up effort, from gene prioritization to drug-target selection. Beyond the methodological contribution, the four-category taxonomy we introduce (direct, indirect, passenger, no effect genes) provides a vocabulary for reasoning about what differential expression results actually mean biologically. We then present conditional differential gene expression (CDGE) analysis as a framework to separate direct from mediated and passenger effects by testing the conditional association between each gene and the trait of interest, given all other genes. For implementation, we perform knockoff-based CDGE analysis by adapting the recently developed GhostKnockoff procedure with lasso regression (Chen *et al.* 2026, He *et al.* 2026). Operating on summary statistics, the method applies to datasets with cell-level records as well as to datasets where only summary statistics are available and provides finite-sample false discovery rate control in direct effect genes identification. We validate CDGE in simulations and across three RNA sequencing datasets of increasing scale. The headline application is a genome-scale perturb-seq experiment (Replogle *et al.* 2022) covering 1284 perturbations: CDGE reduces the number of identified gene–gene connections by approximately 70%, and the retained connections explain about 70% of the connections identified by standard DGE while showing markedly higher enrichment for known protein–protein interactions. Two applications, one in human T-cells from psoriasis patients and one in a Scn8a+/− mouse model of epilepsy, reproduce this pattern: CDGE-identified genes mediate the effects of the large majority of other differentially expressed genes and concentrate biological signal in pathways relevant to disease.

## 2. Methods and materials

We validate CDGE in four settings of increasing scale and decreasing access to ground truth. We first briefly overview the CDGE framework and knockoff-based method, then evaluate it in a controlled simulation that establishes finite-sample false discovery rate control. We next apply knockoff-based CDGE analysis to two single-cell disease studies, one in human T-cells from psoriasis patients (Reynolds *et al.* 2021) and one in a Scn8a+/− mouse model of epilepsy, that test the method on observational human and mouse data with strong prior biological knowledge. Finally, we perform knockoff-based CDGE analysis on genome-scale perturb-seq experiments (Replogle *et al.* 2022), where we identify direct gene–gene regulatory effects across thousands of designed interventions and provide the most stringent external validation against the STRING protein–protein interaction network (Von Mering *et al.* 2005, Szklarczyk *et al.* 2023).

### 2.1. Overview of the method: CDGE analysis via ghostknockoff

Usual DGE analysis infers marginal correlations between the trait of interests (denoted by Y) and the expression level of different genes (denoted by $X_{j}$’s) via testing $H_{M,j}:X_{j}\;⊥\;Y,\;j=1,\ldots ,p$. In this article, we propose the CDGE analysis that investigates the conditional association between each gene and the trait of interests conditioning on all other genes by testing


$$H_{C,j}:X_{j}⊥Y|X_{-j},\;j=1,\ldots ,p,$$

where $X_{-j}$ encodes expression levels of all but the jth genes. That is to say, CDGE analysis aims to identify whether (the distribution of) Y would change when the target gene up-regulates or down-regulates given expression levels of all other genes remain unchanged. As shown in Fig. 1a, when expression levels of all other genes remain unchanged, regulation of the target gene would impact Y only if its direct effect exists. Thus, $H_{C,j}$ depicts the existence of the jth gene’s direct effect (if $H_{C,j}$ is true, the jth gene has no direct effect; if $H_{C,j}$ is false, the jth gene has direct effect) and CDGE analysis can distinguish these direct effects from effects mediated via other genes, providing more information of the underlying biological mechanism. The interpretation of $H_{C,j}$ is similar when we investigate gene–gene connections in Fig. 1b, except that regulation of the measured gene $X_{j}$ would be impacted by Y (indicating whether the perturbation target gene is perturbed or not) if $H_{C,j}$ is false and direct effect from the perturbation target gene to the measured gene exists. In this article, we perform knockoff-based CDGE analysis by adopting the recently developed Ghostknockoff with lasso regression (Chen *et al.* 2026, He *et al.* 2026). Overview of our knockoff-based CDGE analysis is displayed in Fig. 2. It is worth noting that all data information required for our knockoff-based CDGE analysis is encoded into DGE analysis Z-scores and the correlation matrix of expression levels. This property gives our approach the flexibility to be applied to either datasets with cell-level records (as in the psoriasis and epilepsy applications) or with only summary statistics available (as in the perturb-seq application). In addition, by using Z-scores to encode information, we can incorporate existing DGE approach to address any possible batch effects in single cell datasets.

**Figure 2:**
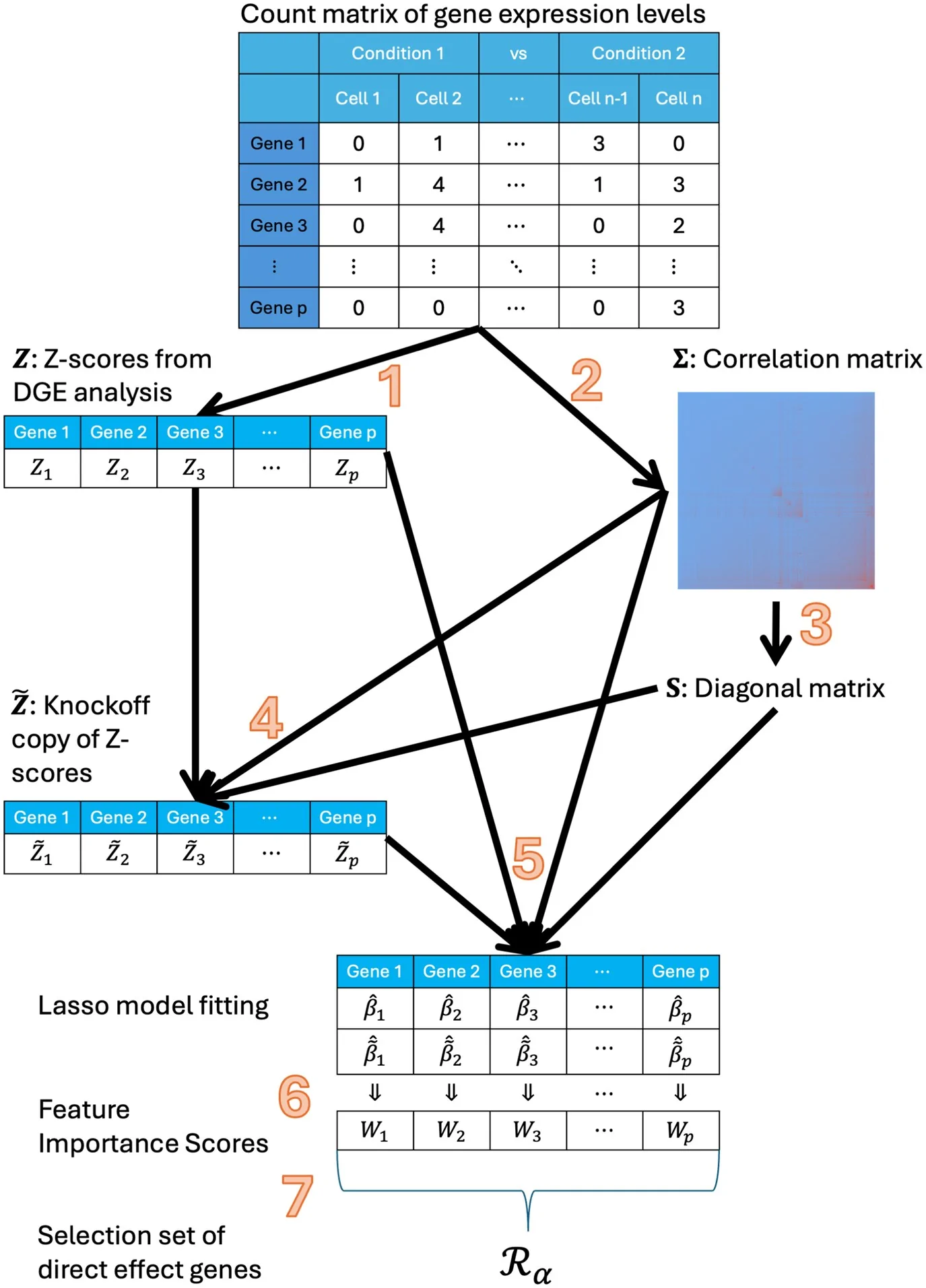
Overview of the knockoff-based CDGE analysis.

### 2.2 Review of existing conditional association analysis

Conditional associations have been extensively studied in literatures of biological data analysis. For example, Ghasemi *et al.* (2021) used the conditional GCTA (COJO) approach to investigate GWAS signals that depicts conditional associations between genetic variants and free thyroxine (FT4), inflammatory bowel disease (IBD) and human height. Gene regulatory network of conditional associations is also investigated in Li *et al.* (2021), Ma *et al.* (2023), Kernfeld *et al.* (2024), and Tejada-Lapuerta *et al.* (2025). Specifically, in investigating conditional association between a large set of biological features (denoted by X**)** and a response of interest (denoted by Y), most of literatures impose a multivariate regression model on the conditional model Y|X. By doing so, null hypothesis of conditional association ($H_{C,j}:X_{j}⊥Y|X_{-j}$) is transferred into null hypothesis of corresponding coefficient $\beta_{j}$ ($H_{j}:\beta_{j}=0$) in the regression model, leading to various parametric tests and variable selection techniques that infer $X_{j}$’s contribution to Y. Examples include t-test, F-test, stagewise regression, LASSO (Tibshirani 1996), and SCAD (Fan and Li 2001). However, most of inferences based on these methods either lack statistical power or do not rigorously control type-I error rate under high-dimensional settings as in transcriptomics studies with thousands of genes. In addition, they could suffer inference bias when the conditional model Y|X is mis-specified (e.g. true model is nonlinear), which is inevitable due to the lack of domain knowledge.

To overcome the aforementioned limitations, the model-X knockoff filter (Candès *et al.* 2018) has been developed recently. Imposing no parametric assumption on the conditional model Y|X, the model-X knockoff filter only requires knowledge of the distribution of X. Its inference is not only finite-sample valid and powerful but also robust to misspecification of the conditional model Y|X. By synthesizing knockoffs $\tilde{X}$ that mimic the dependency structure of X and do not associate with Y beyond X, we use ${\tilde{X}}_{j}$ as a control to depict the association between $X_{j}$ and Y if $X_{j}$ has no direct effect on Y (j=1,…,p). Thus, the model-X knockoff filter can fairly compare the association test statistics of observed features with their knockoff counterparts and identifies false $H_{C,j}$’s with rigorous false discovery rate (FDR) control. Based on this novel idea, a series of knockoff methods have been developed. Specifically, as only false $H_{C,j}$’s correspond to direct effect genes in Fig. 1, model-X knockoff filter can prioritize these genes compared to those marginal association methods.

### 2.3 Conditional differential gene expression (CDGE) analysis via Ghostknockoff

In this article, our CDGE analysis is based on GhostKnockoff approach with lasso regression, which is proposed in He *et al.* (2026) and Chen *et al.* (2026). This allows one to perform CDGE analysis with only summary statistics, defined as Z-scores (or *P* values and directions of differential expressions) from usual DGE analysis and the expression level correlations among the genes. The GhostKnockoff approach is originally derived by He *et al.* (2022) for genome-wide association studies as an analogy of the model-X knockoff filter (Candès *et al.* 2018). This approach can select substantially contributing genetic variants with valid FDR control in the absence of individual data access. On top of that, Chen *et al.* (2026) incorporated the BASIL algorithm (Qian *et al.* 2020) for lasso regression into the GhostKnockoff framework and improved the power. Thus, we adopt the latest GhostKnockoff approach with lasso regression (Chen *et al.* 2026) to our CDGE analysis for RNA sequencing data. The whole implementation workflow is summarized in Fig. 2.

In **step 1**, we perform existing DGE analysis (e.g. DESeq2, EdgeR, and limma) that compares the expression level of each gene across different cell conditions and compute *P* values for all genes (${\mathrm{pval}}_{1},\ldots ,{\mathrm{pval}}_{p}$). We then transfer all *P* values into Z-scores via the inverse cumulative distribution function of standard normal distribution ($\Phi^{-1}(\cdot )$) as


$$Z_{j}={\mathrm{sign}}_{j}\cdot \Phi^{-1}(1-\frac{{\mathrm{pval}}_{j}}{2}),\;j=1,\ldots ,p,$$

where ${\mathrm{sign}}_{j}$ depicts whether the jth gene in cells of contrasted condition is upregulated or downregulated compared to cells of baseline condition.

In **step 2**, we estimate the correlation matrix Σ among expression levels of all genes over control cells or all cells. If the correlation matrix Σ among gene expression levels is known according to domain knowledge or reference studies, we can directly use it. When such existing knowledge is not available, the correlation matrix Σ can be estimated either via the factor model (Leek and Storey 2007, Fan *et al.* 2020) or the shrinkage estimation approach (Schäfer and Strimmer 2005).

Based on correlation matrix Σ, **step 3** is to compute a diagonal matrix $S=\mathrm{diag}(s_{1},\ldots ,s_{p})$ for knockoffs generation under a particular construction regime. Possible regimes include minimizing the mean absolute correlation (Barber and Candès 2015, Candès *et al.* 2018), maximizing the entropy (Gimenez and Zou 2019), and intertwined probabilistic factors decoupling (IPAD; Fan *et al.* 2020).

In our implementation of **step 3**, we adopt the IPAD procedure (Fan *et al.* 2020) to generate knockoffs, which requires to estimate the correlation matrix under the factor model in **step 2**,


X=FL+Δ,

where $F={\left[f_{1},\ldots ,f_{n}\right]}^{T}\in R^{n\times r}$ is the random matrix of unobserved factors, $L={\left[l_{1},\ldots ,l_{p}\right]}^{T}\in R^{p\times r}$ is the deterministic loading matrix, and $\Delta ={\left[\delta_{1},\ldots ,\delta_{n}\right]}^{T}\in R^{n\times p}$ is the matrix of random errors drawn i.i.d. from some parametric models $g_{\eta }(\cdot )$ independent to F. This model is reasonable for gene expression data, where the expression levels are mainly impacted by a small number of technical or biological factors (Buettner *et al.* 2017, Sekula *et al.* 2021). Under the factor model, we suggest using the principal component estimator of correlation matrix computed as follows.

1. Compute all singular values $d_{1}\geq d_{2}\geq \cdots \geq d_{\min\left(n,p\right)}\geq 0$ of X and use the GR criterion of Ahn and Horenstein (2013) to estimate the number of factors as $\hat{r}$.
2. Obtain a rank-$\hat{r}$ approximation of X as ${U\;}_{\hat{r}}\;{D\;}_{\hat{r}}\;V_{\hat{r}}^{T}$ where ${U\;}_{\hat{r}}\in R^{n\times \hat{r}}$ is an orthogonal matrix of left singular vectors, ${D\;}_{\hat{r}}\in R^{\hat{r}\times \hat{r}}$ is a diagonal matrix of the greatest $\hat{r}$ singular values and $V_{\hat{r}}\in R^{p\times \hat{r}}$ is an orthogonal matrix of right singular vectors.
3. Compute $\hat{L}=n^{-\frac{1}{2}}\cdot {D\;}_{\hat{r}}{V\;}_{\hat{r}}^{T}$ and $\hat{\Delta }={\left({\hat{\delta }}_{ij}\right)}_{n\times p}=X-{U\;}_{\hat{r}}\;{D\;}_{\hat{r}}\;V_{\hat{r}}^{T}$.Compute the estimated correlation matrix as

$\hat{\Sigma }={\hat{L}}^{T}\hat{L}+\hat{\Lambda },$

   where $\hat{\Lambda }=\mathrm{diag}({\hat{\lambda }}_{1},\ldots ,{\hat{\lambda }}_{p})=\frac{1}{n}\sum_{i=1}^{n}\mathrm{diag}({\hat{\delta }}_{i1}^{2},\ldots ,{\hat{\delta }}_{ip}^{2})$.

With such an estimated correlation matrix, we can directly compute $S=\hat{\Lambda }$ in **step 3** for knockoffs generation. In Section 3.1.2, our knockoff-based CDGE analysis exhibits its robustness against the estimated $\hat{r}$ and $\hat{\Sigma }$ with empirical FDR control and as high power as possible.

In **step 4**, we synthesize the knockoff copy $\tilde{Z}={\left({\tilde{Z}}_{1},\ldots ,{\tilde{Z}}_{p}\right)}^{T}$ of Z-scores following He *et al.* (2022) that


$$\tilde{Z}=\mathrm{PZ}+E,\;\mathrm{where}\;P=(I-S\Sigma^{-1})\;\mathrm{and}\;E∼N(0,2S-S\Sigma^{-1}S).$$

Here, $\tilde{Z}$ serve as synthetic controls where ${\tilde{Z}}_{j}$ captures all indirect effects from the jth gene ($X_{j}$) to the response Y through other genes. Thus, for those genes without direct effects (whose $H_{C,j}$’s are true), ${\tilde{Z}}_{j}$’s will be similar to $Z_{j}$’s; for those genes with direct effects (whose $H_{C,j}$’s are false), $Z_{j}$’s will dominate ${\tilde{Z}}_{j}$’s by capturing direct effects.

Although it is possible to select direct effect genes with FDR control by directly comparing $Z_{j}$’s and ${\tilde{Z}}_{j}$’s (He *et al.* 2022), doing so may suffer power loss due to noises brought by indirect effects. To circumvent this, we adopt the strategy in He *et al.* (2026) and Chen *et al.* (2026) to fit a lasso regression model and obtain estimates ${\hat{\beta }}_{1},\ldots ,{\hat{\beta }}_{p},{\hat{\tilde{\beta }}}_{1},\ldots ,{\hat{\tilde{\beta }}}_{p}$ in **step 5**, which depict the direct effects of genes and their knockoff copies to the disease, trait of interest or the perturbed gene. Specifically, with $Z,\tilde{Z},\Sigma$, the lasso estimates ${\hat{\beta }}_{1},\ldots ,{\hat{\beta }}_{p},{\hat{\tilde{\beta }}}_{1},\ldots ,{\hat{\tilde{\beta }}}_{p}$ are obtained by solving the optimization problem,


minβ⁡12βTGSβ-1nβT(ZZ∼)+λ‖β‖1,

where $\beta =(\beta_{1},\ldots ,\beta_{p},{\tilde{\beta }}_{1},\ldots ,{\tilde{\beta }}_{p})$, $G_{S}=[\begin{array}{cc} \Sigma & \Sigma -S\\ \Sigma -S & \Sigma \end{array}]$ and λ is computed via the lasso-min method of Chen *et al.* (2026).

As ${\hat{\beta }}_{j}$ captures the direct effect of the jth gene while ${\hat{\tilde{\beta }}}_{j}$ capture the direct effect of the synthetic control that mimics the jth gene, we have (a) for genes without direct effects, ${\hat{\tilde{\beta }}}_{j}$’s will be similar to ${\hat{\beta }}_{j}$’s and (b) for genes with direct effects, ${\hat{\beta }}_{j}$’s will dominate ${\hat{\tilde{\beta }}}_{j}$’s in magnitude. Thus, in **step 6**, we calculate the feature importance score as


$$W_{j}=\mathrm{sign}(\left|{\hat{\beta }}_{j}|-|{\hat{\tilde{\beta }}}_{j}\right|)\cdot \max\left\{\left|{\hat{\beta }}_{j}|,|{\hat{\tilde{\beta }}}_{j}\right|\right\},\;j=1,\ldots ,p,$$

such that $W_{j}$’s tend to be large and positive for direct effect genes and $W_{j}$’s fluctuate around 0 otherwise.

In **step 7**, we apply the knockoff filter (Barber and Candès 2015, Candès *et al.* 2018) to select direct effect genes under the target FDR level α as


$$R_{\alpha }=\{j:W_{j}\geq t_{\alpha }\},\;\mathrm{where}\;t_{\alpha }=\min\left\{t>0|\hat{\mathrm{FDP}}=\frac{1+\#\{j:W_{j}\leq -t\}}{\max\left(1,\#\{j:W_{j}\geq t\}\right)}\leq \alpha \right\}.$$

Theoretical studies in Chen *et al.* (2026) have shown that the selection set of direct effect genes $R_{\alpha }$ has valid control of FDR.

It is worth noting that our knockoff-based CDGE analysis encodes all data information into Z-scores and the correlation matrix of expression levels for inference. This property gives our approach the flexibility to be applied to either datasets with cell-level records (as in the psoriasis and epilepsy applications) or with only summary statistics available (as in the perturb-seq application). As shown in Chen *et al.* (2026), as long as the correlation matrix is well-estimated and the sample size is large enough for approximate normality to hold, the generated knockoff copy $\tilde{Z}$ satisfies the extended exchangeability property (Yu *et al.* 2024) and the obtained set of direct effect genes by the knockoff-based CDGE analysis is equivalent to the second-order knockoffs (Candès *et al.* 2018) which needs access of cell-level records. Thus, our knockoff-based CDGE analysis possesses valid FDR control with respect to $H_{C,j}$’s without substantial power loss. Another benefit of using Z-scores to encode information is that we can incorporate existing DGE approach to address possible batch effects in single cell datasets. By doing so, we do not need to care about batch effects in the generation of $\tilde{Z}$. In contrast, batch effects bring dependency among records of different cells and complicate the generation of cell-level knockoff copies of gene expression levels. This separation of concerns is a structural advantage of the summary-statistics formulation: the upstream DGE model handles cell-level dependencies and confounders, while CDGE operates downstream on Z-scores. Any batch-handling capability of the chosen DGE method (e.g. the design-matrix adjustment in DESeq2, mixed-effects models for repeated measures, or factor-model approaches for unmeasured confounders) is automatically inherited by CDGE, without requiring re-derivation of the knockoff construction for each new design.

#### 2.3.1. Incorporation of multiple knockoffs

Although the knockoff-based CDGE analysis can identify direct effect genes with rigorous guarantee of error rate control, one important flaw is its instability: with randomly generated knockoff copy $\tilde{Z}={\left({\tilde{Z}}_{1},\ldots ,{\tilde{Z}}_{p}\right)}^{T}$ of Z-scores, implementing the procedure twice usually leads to different selection sets. In addition, it may also suffer power loss under low target FDR level α as the filter can only return nonempty selection set with size not smaller than $\alpha^{-1}$, which is called the threshold issue. To improve the stability of the analysis result and address the threshold issue, we incorporate the multiple knockoff implementation (Gimenez and Zou 2019) into our knockoff-based CDGE analysis as follows.

Compared to single knockoff, using multiple knockoffs can reduce the threshold size of nonempty selection set to ${\left(M\cdot \alpha \right)}^{-1}$ and thus improve power when the target FDR level α is low. As demonstrated in the Section 3.1.1, the multiple-knockoffs implementation can also improve the stability of the selection set at minor extra computational cost.

#### 2.3.2. Software implementation

To facilitate public access to knockoff-based CDGE analysis, we have developed the R package “KnockoffCDGE” to provide an integrated implementation. Specifically, the R function “KnockoffCDGE” is the key function to perform analysis with the following input:

1. Z: Z-scores $Z=(Z_{1},\ldots ,Z_{p})$;
2. Sigma: Correlation matrix Σ;
3. M: The number of knockoffs; and
4. n: Sample size;

Please set M = 1 if only single knockoff is used. The output of the R function “KnockoffCDGE” is q-values of all features,


$$q_{j}=\underset{\alpha }{\min}\left\{\alpha :j\in R_{\alpha }\right\},\;j=1,\ldots ,p.$$

Here, $q_{j}$ characterizes the minimum target FDR level at which the jth gene is identified and thus users can obtain $R_{\alpha }=\{j:q_{j}\leq \alpha \}.$

### 2.4 Experiments

#### 2.4.1. Data generation mechanism in simulated experiments

For each replicate, we generate the expression levels of p=500 genes in n=5000 cells as follows.

1. Generate expression levels of indirect effect genes as $X_{\mathrm{indirect},1}={\left(X_{1},\ldots ,X_{20}\right)}^{T}$ where $X_{1},\ldots ,X_{20}$ are i.i.d. random variables from Poisson distribution Pois(1).
2. Generate expression levels of no effect genes as $X_{\mathrm{null},1}={\left(X_{21},\ldots ,X_{80}\right)}^{T}={\left(C_{\mathrm{null},1}+E_{21},\ldots ,C_{\mathrm{null},1}+E_{80}\right)}^{T}$ where $E_{21},\ldots ,E_{80}∼\mathrm{Pois}(1-\rho_{1})$ and $C_{\mathrm{null},1}∼\mathrm{Pois}(\rho_{1})$ are mutually independent with $\rho_{1}=0.7$.
3. Generate expression levels of passenger genes as $X_{\mathrm{passenger},1}={\left(X_{81},\ldots ,X_{90}\right)}^{T}$ from
  Xpassenger, 1=A1(X1⋆⋮X80⋆)+Epassenger, 1,
    where $E_{\mathrm{passenger},1}$ is a 10-dimensional vector of i.i.d. Pois(1) random variables, $A_{1}$ is a random matrix whose entries are i.i.d. samples from Bernoulli distribution Bern(0.2), $X_{i}^{⋆}$’s are independent random variables from $B(X_{i},q_{i})$ with $q_{i}∼\mathrm{Unif}[0,1/4]$ for i=1,…,80.
4. For l=2,…,5,
  - Generate expression levels of indirect effect genes as $X_{\mathrm{indirect},l}={\left(X_{1+90\cdot (l-1)},\ldots ,X_{20+90\cdot (l-1)}\right)}^{T}$ from
    Xindirect,l=Bl(X1+90⋅(l−2)⋆⋮X20+90⋅(l−2)⋆)+Eindirect,l,
        where $E_{\mathrm{indirect},l}$ is a 20-dimensional vector of i.i.d. Pois(1) random variables, $B_{l}$ is a random matrix whose entries are i.i.d. samples from Bernoulli distribution Bern(0.2), $X_{i+90\cdot (l-2)}^{⋆}$’s are independent random variables from $B(X_{i+90\cdot (l-2)},q_{i+90\cdot (l-2)})$ with $q_{i+90\cdot (l-2)}∼\mathrm{Unif}[0,1/4]$ for i=1,…,20.
  - Generate expression levels of no effect genes as $X_{\mathrm{null},l}={\left(X_{21+90\cdot (l-1)},\ldots ,X_{80+90\cdot (l-1)}\right)}^{T}={\left(C_{\mathrm{null},l}+E_{21+90\cdot (l-1)},\ldots ,C_{\mathrm{null},l}+E_{80+90\cdot (l-1)}\right)}^{T}$ where $E_{21+90\cdot (l-1)},\ldots ,E_{80+90\cdot (l-1)}∼\mathrm{Pois}(1-\rho_{l})$ and $C_{\mathrm{null},l}∼\mathrm{Pois}(\rho_{l})$ are mutually independent with $\rho_{l}=\frac{\left(8-l\right)}{10}$.
  - Generate expression levels of passenger genes as $X_{\mathrm{passenger},l}={\left(X_{81+90\cdot (l-1)},\ldots ,X_{90+90\cdot (l-1)}\right)}^{T}$ from
    Xpassenger,l=Al(X1+90⋅(l−1)⋆⋮X80+90⋅(l−1)⋆)+Epassenger,l,
        where $E_{\mathrm{passenger},\;1}$ is a 10-dimensional vector of i.i.d. Pois(1) random variables, $A_{l}$ is a random matrix whose entries are i.i.d. samples from Bernoulli distribution Bern(0.2), $X_{i+90\cdot (l-1)}^{⋆}$’s are independent random variables from $B(X_{i+90\cdot (l-1)},q_{i+90\cdot (l-1)})$ with $q_{i+90\cdot (l-1)}∼\mathrm{Unif}[0,1/4]$ for i=1,…,80.
5. Generate expression levels of direct effect genes as $X_{\mathrm{direct}}={\left(X_{451},\ldots ,X_{500}\right)}^{T}$ from
  Xdirect=Bdirect(X361⋆⋮X380⋆)+Edirect,
    where $E_{\mathrm{direct}}$ is a 50-dimensional vector of i.i.d. Pois(1) random variables, $B_{\mathrm{direct}}$ is a random matrix whose entries are i.i.d. samples from Bernoulli distribution Bern(0.2), $X_{i}^{⋆}$’s are independent random variables from $B(X_{i},q_{i})$ with $q_{i}∼\mathrm{Unif}[0,1/4]$ for i=361,…,380.

Based on expression levels $X=(X_{1},\ldots ,X_{500})$, the response Y is generated from the logistic model


$$\mathrm{logit}\{P(Y=1|X)\}=\beta_{0}+\sum_{j=451}^{500}\beta_{j}X_{j}.$$

Here, $\beta_{451},\ldots ,\beta_{500}$ are i.i.d. samples from the normal distribution N(0,0.02) and Y denotes an indicator of whether the cell comes from the disease group. By doing so, we simulate all four types of genes with respect to Y (direct effect genes, indirect effect genes, passenger genes, and no effect genes). For example, $X_{451},\ldots ,X_{500}$ correspond to direct effect genes and $X_{361},\ldots ,X_{380}$ are indirect effect genes which affect both direct effect genes and passenger genes $X_{441},\ldots ,X_{450}$.

#### 2.4.2. Adjusting for CDGE-identified genes in the DGE analysis

Based on Z-scores Z and correlation matrix Σ, our knockoff-based CDGE analysis manages to identify a set of genes $R_{\alpha }$ where on average, there are 100⋅(1-α)% genes with truly direct effects. To validate that these identified direct effect genes mediate the effects of the remaining differentially expressed genes, we adjust for CDGE-identified genes and perform DGE analysis of all other genes with detailed procedures as follows.

Multiple-Knockoffs-based CDGE analysis

1. Obtain Z-scores via DGE analysis $Z=(Z_{1},\ldots ,Z_{p}).$
2. Estimate the correlation matrix Σ among gene expression levels.
3. Compute a diagonal matrix $S=\mathrm{diag}(s_{1},\ldots ,s_{p})$ for knockoffs generation based on Σ and the number of knockoff copies M (when adopting the IPAD procedure to generate knockoffs with Σ estimated under the factor model, S is computed as $\hat{\Lambda }$ for all M≥1).
4. Synthesize knockoff copies ${\tilde{Z}}^{\left(1\right)},\ldots ,{\tilde{Z}}^{\left(M\right)}$ via of Z-scores following He et al. (2022) that
  (Z∼(1)⋮Z∼(M))=PZ+E,
     where
  P=(I-SΣ-1⋮I-SΣ-1), E∼N(0,V)
     and
  $$V=(\begin{array}{cccc} 2S-S\Sigma^{-1}S & S-S\Sigma^{-1}S & \cdots & S-S\Sigma^{-1}S\\ S-S\Sigma^{-1}S & 2S-S\Sigma^{-1}S & \cdots & S-S\Sigma^{-1}S\\ \vdots & \vdots & \ddots & \vdots \\ S-S\Sigma^{-1}S & S-S\Sigma^{-1}S & \cdots & 2S-S\Sigma^{-1}S \end{array}).$$
     Specifically, our implementation adopts the efficient algorithm (Algorithm 2) of Yu *et al.* (2024). For convenience, we also denote $Z={\tilde{Z}}^{\left(0\right)}$.
5. Compute the lasso estimate $\hat{\beta }$ by solving the optimization problem,
  minβ⁡12βTGSβ-1nβT(Z∼(0)⋮Z∼(M))+λ‖β‖1,
     where $\beta =(\beta_{1}^{\left(0\right)},\ldots ,\beta_{p}^{\left(0\right)},\ldots ,\beta_{1}^{\left(M\right)},\ldots ,\beta_{p}^{\left(M\right)})$, $G_{S}=1_{\left(M+1)\times (M+1\right)}⊗(\Sigma -S)+I_{\left(M+1)\times (M+1\right)}⊗S$, ⊗ is the Kronecker product, $1_{\left(M+1)\times (M+1\right)}$ is the (M+1)×(M+1) matrix of 1’s, $I_{\left(M+1)\times (M+1\right)}$ is the (M+1)×(M+1) identity matrix and λ is computed via the lasso-min method of Chen *et al.* (2026).
6. Calculate the feature importance score as
  $$\kappa_{j}=\underset{0\leq m\leq M}{\mathrm{argmax}}\left|{\hat{\beta }}_{j}^{\left(m\right)}\right|,$$
   
  $$\tau_{j}=|{\hat{\beta }}_{j}^{\left(\kappa_{j}\right)}|-\mathrm{median}\{{\hat{\beta }}_{j}^{\left(m\right)}|m\neq \kappa_{j}\},\;j=1,\ldots ,p,$$
  such that $\kappa_{j}$’s are more likely to be zero and $\tau_{j}$’s tends to be large for direct effect genes, while $\kappa_{j}$’s are uniformly distributed in {0,1,…,M} otherwise.
7. Apply the filter (Gimenez and Zou 2019) to select direct effect genes under the target FDR level α as
  $$R_{\alpha }=\{j:\kappa_{j}=0,\tau_{j}\geq t_{\alpha }\},$$
  where$\;t_{\alpha }=\min\left\{t>0|\hat{\mathrm{FDP}}=\frac{\frac{1}{M}\cdot (1+\#\{j:\kappa_{j}\neq 0,\tau_{j}\geq t\})}{\max\left(1,\#\{j:\kappa_{j}=0,\tau_{j}\geq t\}\right)}\leq \alpha \right\}.$

DGE analysis adjusting for CDGE-identified genes

1. Input: Z-scores $Z=(Z_{1},\ldots ,Z_{p})$, correlation matrix Σ and the selection set R.
2. Extract the subvector $Z_{R}$ of Z corresponding to all genes in R. Similarly, we can extract subvector $Z_{R^{c}}$ corresponding to all genes not in R and submatrices $\Sigma_{R,R}$, $\Sigma_{R^{c},R^{c}}$ and $\Sigma_{R^{c},R}$.
3. Compute $Z_{R^{c}|R}=Z_{R^{c}}-\Sigma_{R^{c},R}\Sigma_{R,R}^{-1}Z_{R}\;\mathrm{and}\;\Sigma_{R^{c}|R}=\Sigma_{R^{c},R^{c}}-\Sigma_{R^{c},R}\Sigma_{R,R}^{-1}\Sigma_{R^{c},R}^{T}.$
4. For all $j\in R^{c}$, compute the DGE p-value after adjusting as ${\mathrm{pval}}_{j}^{*}=2\cdot \{1-\Phi (\left|Z_{j}^{*}\cdot {\left(\sigma_{j}^{*}\right)}^{-1/2}\right|)\}$ where $Z_{j}^{*}$ is the corresponding element in $Z_{R^{c}|R}$ and $\sigma_{j}^{*}$ is the corresponding diagonal element in $\Sigma_{R^{c}|R}$.
5. Output: Sets of genes $R^{*}$ that remains significantly differentially expressed under the prespecified target FDR level with BH correction.

#### 2.4.3. Additional experiment details of the epilepsy dataset

The mice experiment that collects the epilepsy dataset is performed through dissection of four heterozygous (Scn8a+/−) mice and four wild-type (Scn8a+/+) mice as controls (RRID: IMSR_JAX: 003798). All mice are 56–57 days old when their brains are extracted, where the cuts are positioned at the Bregma coordinates 1.045 mm to −1.055 mm and subcortical regions below the corpus callosum are removed. The extracted tissues are then stored in MACS Tissue Storage Solution with 45 μM Actinomycin D. After isolating nuclei using MACS sorting, we fix brain nuclei of mice using Parse biosciences fixation kit, run them thought the Parse biosciences WT barcoding kit (capacity: 100k cell/nuclei). We then sequence cDNA of different brain cells under Seqmatic Novoseq X Plus Run, which returns Fastq files. For statistical inference in R, we finally pass the Fastq files through Parse Pipeline V1.1.0 to demultiplex the raw reads into a RDS file.

## 3. Results

### 3.1. Simulation verification that CDGE analysis identifies direct effects

To validate our statement that CDGE analysis can identify direct effect genes, we conduct a simulated experiment with p=500 genes, n=5000 cells, and 1000 replicates. Specifically, expression levels of these p=500 genes are generated from the hierarchical model as visualized in Fig. 3a while the response Y is generated from the logistic model. Details of the data generation mechanism are provided in Section 2.4.1. To mimic the complexity of biological pathways, we set five layers of indirect effect genes. While existing DGE tests are anticipated to identify direct effect genes, indirect effect genes, and passenger genes (respectively colored black, red, and purple in Fig. 3a), we expect CDGE analysis can narrow down the focus to identify direct effect genes ($X_{451},\ldots ,X_{500}$).

**Figure 3:**
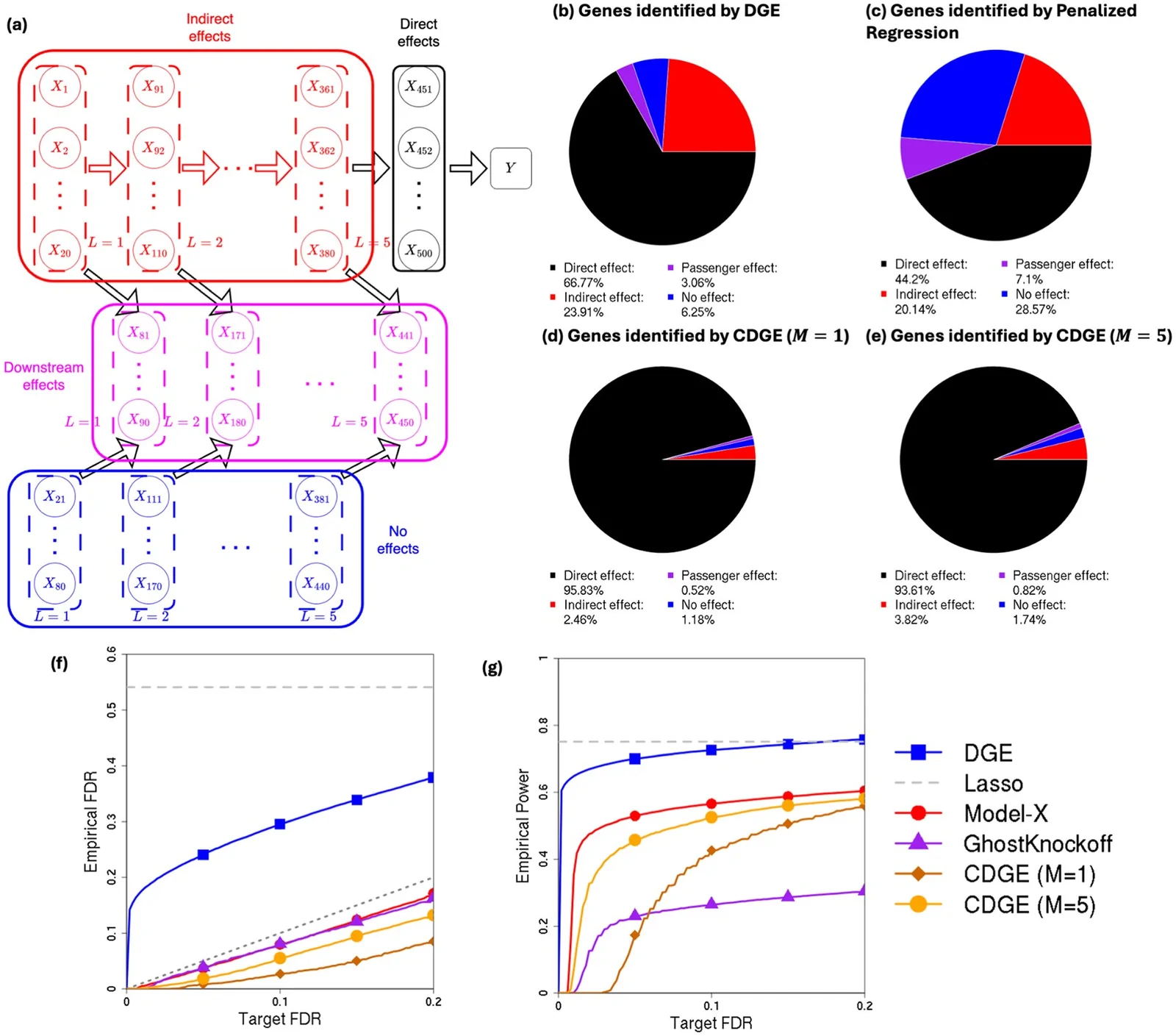
(a) Graphical illustration of the hierarchical model depicting the data generation mechanism of p=500 genes and the response in simulated experiments. (b) Average proportions of different types of DGE-identified genes under the FDR level 0.10 with BH correction. (c) Average proportions of different types of genes identified as direct effect genes under penalized regression. (d) Average proportions of different types of CDGE-identified genes under the FDR level α=0.10 with the single-knockoff implementation (M=1). (e) Average proportions of different types of CDGE-identified genes under the FDR level α=0.10 with the multiple-knockoffs implementation (M=5). (f) Empirical FDR of DGE, penalized regression, model-X knockoffs, the original Ghostknockoff approach and knockoff-based CDGE analysis (M=1 and 5) in identifying direct effect genes over all target FDR levels α∈[0,0.2]. (g) Empirical power of DGE, penalized regression, model-X knockoffs, the original Ghostknockoff approach and knockoff-based CDGE analysis (M=1 and 5) in identifying direct effect genes over all target FDR levels α∈[0,0.2].

We perform DGE analysis on this data to compare gene expression levels between cells in the disease group (Y=1) and the control group (Y=0) via the Wilcoxon rank-sum test and we compute the Z-score of the jth gene as $Z_{j}$ for j=1,…,p. We first display in Fig. 3b the average proportions of different types of DGE-identified genes under the FDR level 0.10 with the Benjamini-Hochberg (BH; Benjamini and Hochberg 1995) correction. Although DGE analysis manages to identify a lot of genes on the pathways (direct effect genes and indirect effect genes), direct effect genes account for only 66.77% of DGE-identified genes. That is to say, DGE analysis cannot distinguish direct effect genes from other genes with differential expression (indirect effect genes and passenger genes account for 23.91% and 3.06%, respectively). To account for correlations among expression levels, penalized regression is another popular category of approaches for direct effect genes identification. However, when we apply the logistic regression with lasso penalty, only 44.20% of identified genes are direct effect genes as shown in Fig. 3c. In short, neither DGE analysis nor penalized regression provide FDR-controlled detection of direct effects.

We implement our knockoff-based CDGE analysis to identify direct effect genes at the same target FDR level α=0.10. Here, we consider both the single-knockoff implementation (M=1) and multiple-knockoffs implementation with M=5 knockoff copies of Z-scores. To validate that CDGE-identified genes mainly correspond to those of direct effects, we present the average proportions of different types of genes in these CDGE-identified genes over 1000 replicates in Fig. 3d and e. We find that although both the knockoff-based CDGE analysis and DGE analysis only identify several no effect genes, CDGE analysis prioritizes direct effects, no matter whether M=1 or M=5. Specifically, over 1000 replicates, the average proportions of CDGE-identified genes without direct effects are only 4.17% (M=1) and 6.39% (M=5) while there are 90% among all genes, suggesting that CDGE analysis does identify direct effect genes with valid FDR control. Such an FDR control remains valid throughout all target FDR levels α∈[0,0.2] as displayed in Fig. 3f, in comparison to the inflated FDR of both DGE analysis and penalized regression.

However, there exhibits substantial power gap between the single-knockoff implementation (M=1) and multiple-knockoffs implementation (M=5), especially when the target FDR level α is in [0,0.1] where the threshold issue (detailed in Section 2.3.1) is most severe for M=1. That is to say, using multiple-knockoffs implementation with M=5 knockoff copies can substantially address the threshold issue and maintain high power. Further comparisons between the single-knockoff implementation and multiple-knockoffs implementation with different M are made in the Section 3.1.1.

We also compare our knockoff-based CDGE analysis with the model-X knockoffs (Candès *et al.* 2018) and the original Ghostknockoff approach (He *et al.* 2022) that only use Z-scores and its knockoff copies for knockoff inference. On the one hand, because our knockoff-based CDGE analysis is a summary-statistics method, comparing with the model-X knockoffs investigates the power loss to apply a summary-statistics method when cell-level data are available. On the other hand, comparing with the original Ghostknockoff approach helps to understand the role of lasso regression in distilling direct effects from Z-scores. For fair comparison, both comparing methods are implemented with multiple knockoffs (M=5). Specifically, in the implementation of model-X knockoffs, we adopt the second-order construction of Candès *et al.* (2018) in synthesizing knockoff copies of gene expression levels X and fit a logistic regression model with lasso penalty to obtain estimates for inference. As shown in Fig. 3g, we find that our knockoff-based CDGE analysis substantially outperforms the original Ghostknockoff approach. This suggests that the lasso regression manages to distill direct effects from noise and accurately pinpoint direct effect genes. In addition, our knockoff-based CDGE only suffers minor loss with respect to the model-X knockoffs. That is to say, although the knockoff-based CDGE analysis is implemented on the summary statistics Z, it does not miss substantial information related to genes’ direct effects.

#### 3.1.1. Multiple-knockoffs implementation is more stable

As demonstrated in the Section 2.3.1, theoretical advantages of multiple-knockoffs implementation include more stable power against threshold issue under low target FDR level and more stable selection sets across independent runs on the same dataset. To empirically verify such advantages, we compare the performance of the single-knockoff implementation (M=1) and the multiple-knockoffs implementations with M=2, 3, 4, 5, 10, 50, 100 on 1000 simulated datasets. As displayed in Fig. S1a and b, available as supplementary data at *Bioinformatics* online, all implementations of the knockoff-CDGE analysis under comparison provide valid FDR control and the empirical power under the same target FDR level increases as the number of knockoff copies M grows from 1 to 5. The power gain from increasing M; however, becomes neglectable when M=5. If M exceeds 5, the power under low target FDR level keeps growing due to milder threshold issue. However, such a gain comes at the cost of lower power under higher target FDR level, because more knockoff copies lead stronger noise in detecting medium-to-weak signals. To qualify the stability of the selection sets obtained under different implementations, we perform ten independent runs of all considered implementations (M=1, 2, 3, 4, 5, 10, 50, 100) on each simulated dataset, compute the selection sets $R^{\left[1\right]},\ldots ,R^{\left[10\right]}$ under the target FDR level α=0.1 and compute the stability index among $R^{\left[1\right]},\ldots ,R^{\left[10\right]}$ as


SI=(102)-1⋅∑1≤a<b≤10#(R[a]∩R[b])max⁡(1,#(R[a]∪R[b])).

The larger SI is, the more stable the selection set is. Fig. S1c, available as supplementary data at *Bioinformatics* online, demonstrates that the multiple-knockoffs implementation with M=5 can improve the stability of the selection set, with minor extra computational cost displayed in Fig. S1d, available as supplementary data at *Bioinformatics* online. However, when M exceeds 5, the gain in stability vanishes while the computational cost increases (because there will be unavoidably more nonzero estimates in the lasso regression). In summary, multiple-knockoffs implementation with larger M does have more stable power and selection sets, and we can achieve nearly-optimal performance with moderate M. Based on these results, we mainly use the multiple-knockoffs implementation (M=5) in the following experiments.

#### 3.1.2. Robustness against estimated number of factors and Σ

Similar to the model-X knockoff and original GhostKnockoff approach, our knockoff-based CDGE analysis is derived under the known correlation matrix Σ. However, in practice, such a quantity is usually not provided in advance and warrants estimation. This posts an question of practice use that whether the FDR control remains valid under an estimated Σ. With the focus on the correlation matrix estimation under the factor model, the estimation error of Σ is mainly attributed to the estimation of the number of factors r. To investigate the robustness of our knockoff-based CDGE analysis, we compare our knockoff-based CDGE analysis (M=5) with estimated $\hat{r}$ against with fixed r on 1000 simulated datasets.

We first visualize the empirical distribution of the estimated $\hat{r}$ in Fig. S2a, available as supplementary data at *Bioinformatics* online, where most of estimated $\hat{r}$ take values 5, 6, and 7. Thus, we consider comparison against our knockoff-based CDGE analysis (M=5) with fixed r=1,…,10. It is expected that using estimated correlation matrix with an underestimated r cannot fully capture dependency among genes and would inflate the FDR, while using estimated correlation matrix with an overestimated r leads to smaller S matrix, more similarity between Z-scores and their knockoff copies and finally lower power in distinguishing direct effect genes. Figure S2b–e, available as supplementary data at *Bioinformatics* online, empirically verifies such a theoretical expectation. With the fixed r≤4, running our knockoff-based CDGE analysis procedure have inflated FDR over the target levels 0.10 and 0.20. As the fixed r grows, the FDR is controlled at the cost of decreasing power. In contrast, as the estimated $\hat{r}$ mainly take values 5, 6, and 7, our knockoff-based CDGE analysis maintains FDR control empirically with as high power as possible. This depicts the robustness of our knockoff-based CDGE analysis in FDR and power against the estimated $\hat{r}$ and $\hat{\Sigma }$.

#### 3.1.3. Other sensitivity analyses

We also performed multiple sensitivity analyses to investigate the performance of our knockoff-based CDGE analysis (M=5) under different scenarios. Under the same data generation mechanism described in Section 2.4.1, Fig. S3, available as supplementary data at *Bioinformatics* online, shows that our knockoff-based CDGE analysis (M=5) maintains valid FDR control even when the sample size (n) and the number of genes (p) are comparable, while the power is mainly determined by the sample size. It is interesting that with the same sample size, more genes (larger p) would lead to stronger shrinkage of lasso regression and thus lower power. We present the empirical performance in Fig. S4, available as supplementary data at *Bioinformatics* online, when the number of direct effect genes differs. With FDR controlled as expected, we find our method more favored in the sparse setting. When there are only 10 direct effect genes, the strength of each signal is stronger, and our method has the largest power (under the target FDR level α=0.1). As the number of direct effect genes grows, signals become denser but weaker, making it harder for our filter to distinguish the true signals from noises (Yu *et al.* 2024). Moreover, via Figs S5 and S6, available as supplementary data at *Bioinformatics* online, we verify our knockoff-based CDGE analysis’s FDR control against different signal strengths and collinearity levels, and its power increases as signal strength grows or collinearity weakens. We note the conservative nature of the knockoff-based CDGE analysis under severe collinearity ($\lambda_{E}=1/2$ in Fig. S6, available as supplementary data at *Bioinformatics* online). This is because the diagonal matrix S is smaller under stronger collinearity, making Z-scores and their knockoff copies more similar and harder to distinguish. Thus, for direct effect genes with stronger collinearity, it is less likely to have a large and positive $W_{j}$ (or $\kappa_{j}=0$ and large $\tau_{j}$ under multiple-knockoffs implementation) and less likely to be selected. In summary, our knockoff-based CDGE analysis provides consistent FDR throughout various scenarios.

### 3.2. Applications to psoriasis dataset

We consider the scRNA-seq dataset collected by Reynolds *et al.* (2021) in the study of inflammatory skin diseases. With the focus on psoriasis, a chronic autoimmune disease leading to abnormal patches on skin, we extract gene expression records of 120 309 skin cells from three psoriasis patients (denoted as P1, P2, and P3). Note that for each patient, epidermis and dermis cells in both lesional and non-lesional regions are collected and sequenced. Expression levels of 28 383 genes are measured by counts of corresponding mRNA over all cells. Based on the knowledge that psoriasis is a well-understood homogeneous disease with highly successful therapeutics around the T-cell axis, we focus our analysis on 23 090 T-cells. The numbers of different types of T-cells from different patients are summarized in Fig. 4a. For numerically stable inference and better biological relevance, we filter out genes that pass the Cook’s distance threshold or express in less than 1% cells, leading to 9661 genes remaining in our analysis. (Here, genes that pass the Cook’s distance threshold would return NA in DGE p values, while the approximate normality is not expected to hold for the genes with an excessive number of zeros.)

**Figure 4:**
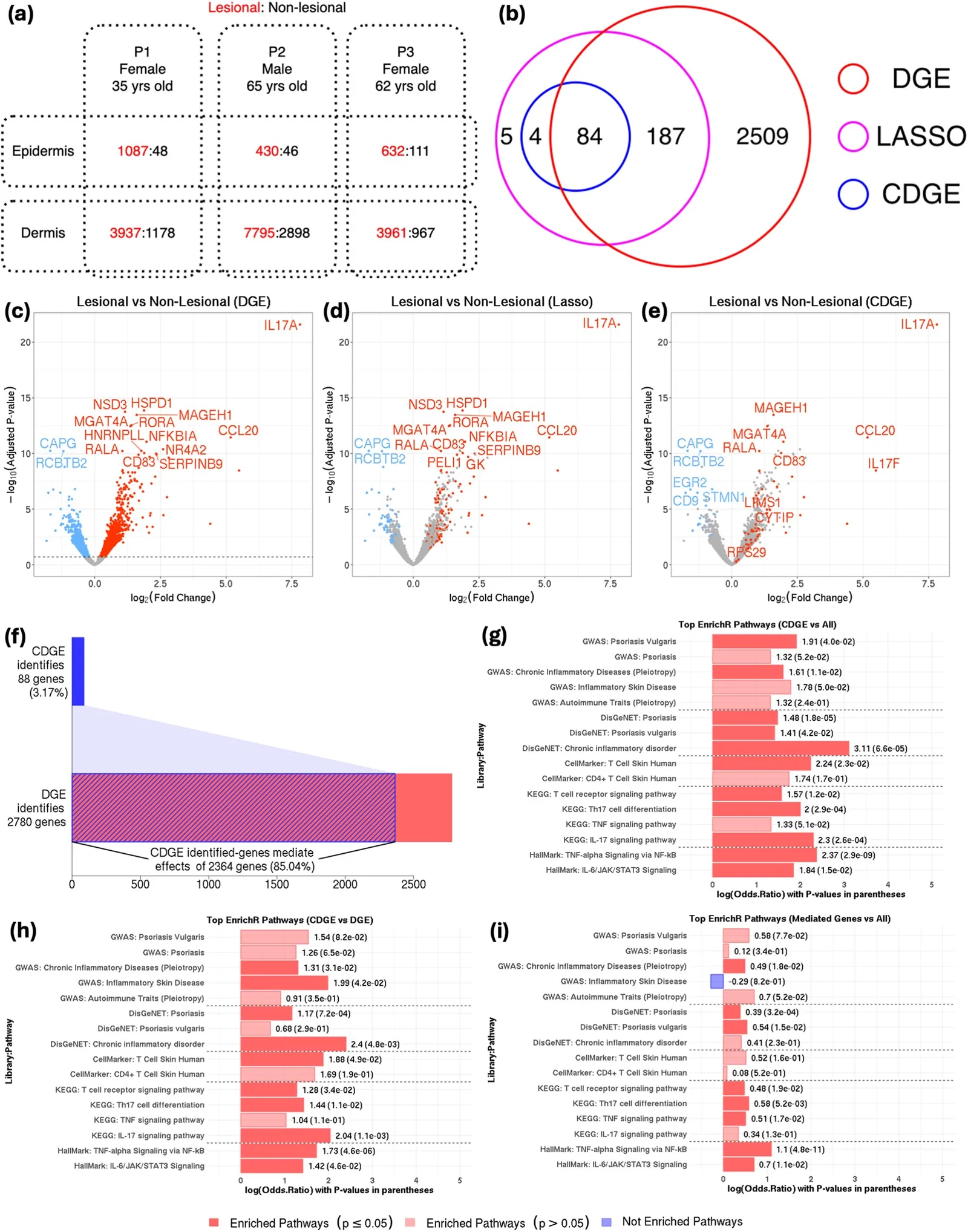
(a) The number of epidermis and dermis T-cells in both lesional and non-lesional regions from different patients. (b) The Venn diagram of DGE-identified genes (under the target FDR level 0.20 with BH correction), lasso-identified genes and CDGE-identified genes (under the target FDR level α=0.20). (c) Volcano plot of DGE analysis, where DGE-identified genes (under the target FDR level 0.20 with BH correction) are highlighted in red (upregulation) and blue (downregulation). (d) Volcano plot of lasso regression, where lasso-identified genes are highlighted in red (upregulation) and blue (downregulation). (e) Volcano plot of knockoff-based CDGE analysis, where CDGE-identified genes (under the target FDR level α=0.20) are highlighted in red (upregulation) and blue (downregulation). (f) Comparison of the number of CDGE-identified genes, the number of DGE-identified genes and the number of genes whose effect is mediated by CDGE-identified genes. (g) Enrichment analysis of CDGE-identified genes in biological pathways related to psoriasis, inflammatory diseases, autoimmune traits and skin T-cells (compared to all genes). (h) Enrichment analysis of CDGE-identified genes in biological pathways related to psoriasis, inflammatory diseases, autoimmune traits and skin T-cells (compared to DGE-identified genes). (i) Enrichment analysis of the remaining DGE-identified genes after excluding CDGE-identified genes in biological pathways related to psoriasis, inflammatory diseases, autoimmune traits and skin T-cells (compared to all genes).

To begin with, we perform the pseudo-bulk DGE analysis using DESeq2 to investigate the marginal association between gene expression levels and the lesional state of cells, adjusting for their origins (P1, P2, or P3) and anatomic states (dermis or epidermis). We identify 2780 differentially expressed genes under the target FDR level 0.20 with the BH correction. Volcano plot of the DGE analysis is provided in Fig. 4c and details of these differentially expressed genes are in Supplementary Table S1, available as supplementary data at *Bioinformatics* online. As differentially expressed genes can be either with direct effects, with indirect effects or with passenger effects, we cannt tell which genes have direct effects from Fig. 4c. Genes with smallest *P* values are possible to be those with indirect effects or passenger effects. For example, among the identified differentially expressed genes, IL17A, IL17F, CCL20, IL23R, and IL22 are known to be on biological pathways of psoriasis in an extensive literature, and the corresponding targeted gene therapies have been shown to be effective (Furue *et al.* 2020, Elnabawi *et al.* 2021, Ghoreschi *et al.* 2021, Reynolds *et al.* 2021). However, they may not be the top signals with smallest *P*-values in the DGE analysis, and it is difficult to determine whether their effects are direct effects. For example, IL23R and IL22 have the 74th and 699th smallest *P* values, respectively. Although solely performing the lasso regression manages to identify 280 genes (shown in Fig. 4d and Table S1, available as supplementary data at *Bioinformatics* online) that are likely to have direct effects, we do not have rigorous guarantee on the proportion of false discoveries among these genes.

We conduct the knockoff-based CDGE analysis on DGE Z-scores and expression level correlation matrix Σ of all genes to distill direct effects with FDR control. Considering that cells from the same patients are not independent, we estimate the correlation matrix Σ under a factor model (Leek and Storey 2007, Fan *et al.* 2020) with the estimated number of factors $\hat{r}=8$. To account for the randomness of knockoff generation, we perform 100 independent runs of the knockoff-based CDGE analysis and summarize the 100 obtained selection sets under the target FDR level α=0.20 via the inclusion-rate based approach [Algorithm 2 of Dai *et al.* (2023)]. Figure 4e highlights all 88 CDGE-identified genes (under the target FDR level α=0.20) in the volcano plot, whose details can also be found in Table S1, available as supplementary data at *Bioinformatics* online. As shown by the Venn diagram in Fig. 4b, all CDGE-identified genes are selected by the sole lasso regression, implying that the proportion of direct effect genes in the remaining 192 genes (identified by lasso but not CDGE) should be under 80%. Specifically, genes IL17A, IL17F, CCL20, and IL23R are significant in the knockoff-based CDGE analysis, suggesting that they are more likely to have direct effects.

To validate that these identified direct effect genes mediate the effects of the remaining differentially expressed genes, we adjust for CDGE-identified genes and perform DGE analysis of all other genes, with detailed procedures in Section 2.4.2. After adjusting for 88 CDGE-identified genes, only 416 differentially expressed genes maintain their statistical significance in the DGE analysis (under the target FDR level 0.20 with the BH correction). This implies that the other 2364 differentially expressed genes have either direct effects or effects mediated by CDGE-identified genes (as shown in Fig. 4f), which is consistent with the statement that most CDGE-identified genes are truly direct effect genes. We note that with rigorous FDR control, the knockoff-based CDGE analysis is conservative in detecting genes with evidence strength at the boundary level, which unavoidably leads to false negatives (i.e. direct effect genes mediating the effect of the 416 remaining differentially expressed genes). In addition, conditioning on a large covariate set reduces statistical power generally, so the strongest causal-mediation interpretation requires further validation, the result we report should be read as evidence that CDGE-identified genes capture the bulk of the trait-relevant signal in this dataset, rather than as a definitive causal claim.

As empirical evidence shows that most gene effects on psoriasis are mediated by the CDGE-identified genes, these genes should provide more information of psoriasis-related biological processes. To verify this, we perform enrichment analysis using multiple biological process pathways related to psoriasis, inflammatory diseases, autoimmune traits, and skin T-cells. These pathways come from different libraries of the “Enrichr” database (Chen *et al.* 2013, Kuleshov *et al.* 2016, Xie *et al.* 2021) and their detailed information is provided in Table S2, available as supplementary data at *Bioinformatics* online. Figures 4(g)–(h) present the enrichment of CDGE-identified genes over psoriasis-related pathways, in comparison against all genes and DGE-identified genes, respectively. In general, CDGE-identified genes are enriched in all relevant pathways. For example, in addition to IL17A, IL17F, and IL23R, our CDGE identifies the fundamental gene NFKBIA for Th17 cells differentiation, which activates cytokine receptors (Park *et al.* 2014). Another CDGE-identified gene TNFAIP8 is also known to be involved in the dysregulation of the production of cytokine TNFα, which associates with the development of psoriasis (Bradley 2008, Niture *et al.* 2019). In contrast, differentially expressed genes STAT3 and JAK1 used as drug targets are not identified in CDGE analysis, suggesting these genes are likely to have no direct effects to psoriasis. Although we can expect there remain several direct effect genes unidentified, effects of these false negatives should be neglectable as shown in Fig. 4i, where the differentially expressed genes not identified by CDGE have lower enrichment level than CDGE-identified genes in all relevant biological process pathways.

### 3.3. Applications to epilepsy dataset

We also apply the knockoff-based CDGE analysis to a single-nucleus RNA-seq (snRNA-seq) dataset collected in investigation of biological mechanisms underlying epilepsy progression. Epilepsy is a type of non-communicable neurological disorder defined by the predisposition for recurrent, unprovoked seizures (Srivastava *et al.* 2021, Knowles *et al.* 2022). This snRNA-seq dataset is collected using a genetic mouse model of generalized epilepsy, *Scn8a*+/−. These mice express a loss of function mutation in *Scn8a*, which encodes the voltage-gated sodium channel alpha subunit (${Na}_{v}1.6$). Such a loss of function mutation leads to spontaneous, progressive absence seizures (Papale *et al.* 2009, Makinson *et al.* 2017, Knowles *et al.* 2022). To understand biological pathways of epilepsy progression in the *Scn8a*+/− model, we sequence expression levels of 57 010 genes over 63 141 cells from 8 mice, including 4 *Scn8a*+/− mice and 4 *Scn8a*+/+ littermate control mice. Details of our biological experiment are provided in the Section 2.4.3. Expression levels of all genes are measured by counts of corresponding mRNA molecules over all cells. Motivated by the previous discovery (Knowles *et al.* 2022) that seizures in *Scn8a*+/− mice induce activity-dependent proliferation and maturation of oligodendrocyte precursor cells (OPCs), as well as increase callosal myelination which in turn, promotes further progression of epilepsy, we focus on differentially expressed genes in OPCs (1905 in total). Genotypes, sex of these eight mice and the number of their OPCs is summarized in Fig. 5a.

**Figure 5:**
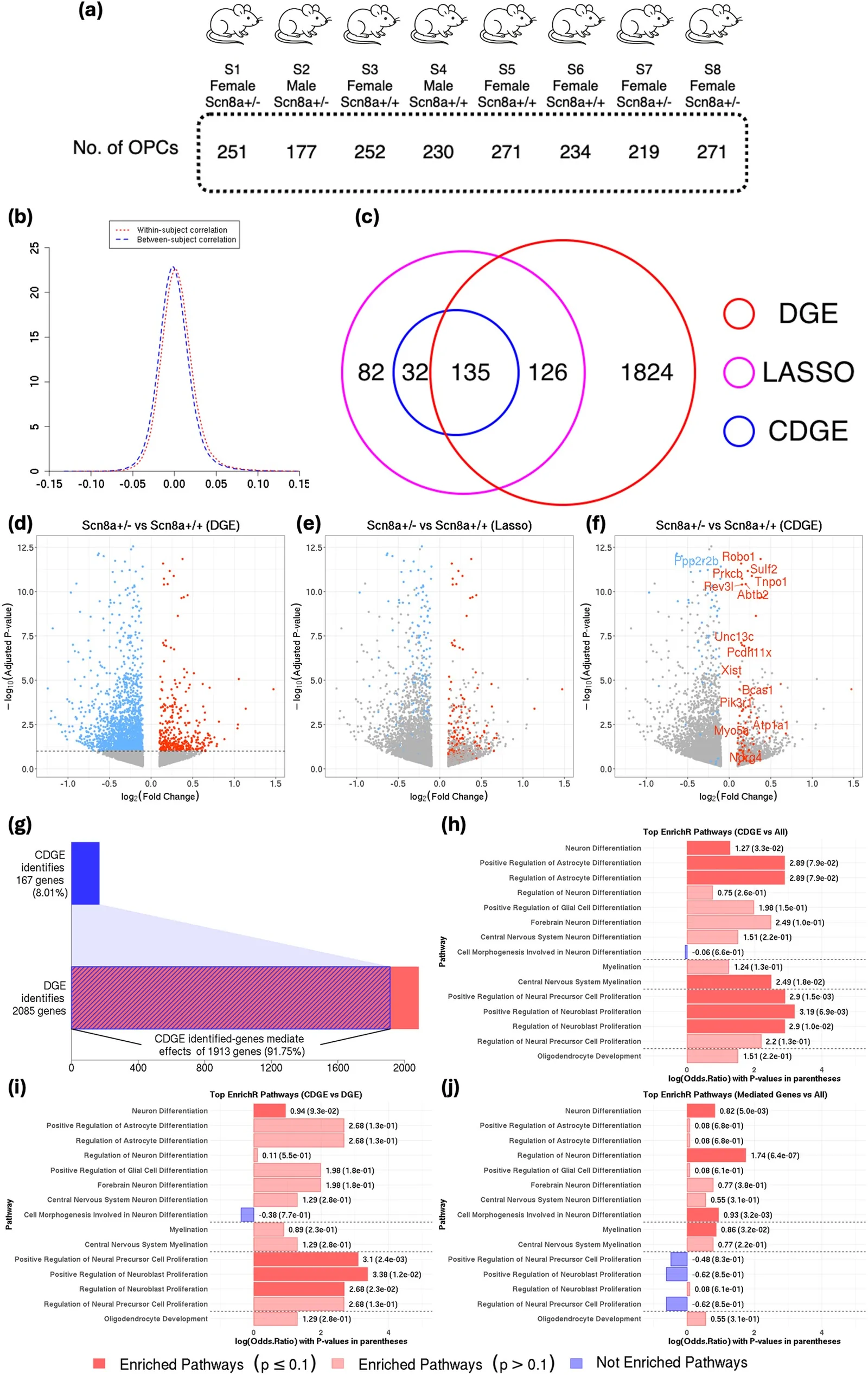
(a) Genotype, sex and the number of OPCs for each mouse. (b) Kernel density estimates of the distribution of gene expression correlation between cells from the same mouse (red dotted line) or between cells from different mice (blue dashed line). (c) The Venn diagram of DGE-identified genes (under the target FDR level 0.10 with BH correction), lasso-identified genes and CDGE-identified genes (under the target FDR level α=0.10). (d) Volcano plot of DGE analysis, where DGE-identified genes (under the target FDR level 0.10 with BH correction) are highlighted in red (upregulation) and blue (downregulation). (e) Volcano plot of lasso regression, where lasso-identified genes are highlighted in red (upregulation) and blue (downregulation). (f) Volcano plot of knockoff-based CDGE analysis, where CDGE-identified genes (under the target FDR level α=0.10) are highlighted in red (upregulation) and blue (downregulation). (g) Comparison of the number of CDGE-identified genes, the number of DGE-identified genes and the number of genes whose effect is mediated by CDGE-identified genes. (h) Enrichment analysis of CDGE-identified genes in biological pathways related to psoriasis, inflammatory diseases, autoimmune traits and skin T-cells (compared to all genes). (i) Enrichment analysis of CDGE-identified genes in biological pathways related to psoriasis, inflammatory diseases, autoimmune traits and skin T-cells (compared to DGE-identified genes). (j) Enrichment analysis of the remaining DGE-identified genes after excluding CDGE-identified genes in biological pathways related to psoriasis, inflammatory diseases, autoimmune traits and skin T-cells (compared to all genes).

For numerical stability of analysis, we filter out all genes with no more than 5% nonzero counts among the 1905 OPCs (here, the approximate normality is not expected to hold for the genes with an excessive number of zeros.). There remain 6163 genes in our analysis. Before we perform the DGE analysis, we first investigate whether cells from the same mice are correlated or not. As shown in Fig. 5b, gene expression correlation between cells from the same mouse follows the same distribution as the gene expression correlation between cells from different mice, suggesting that the within-subject dependency of gene expression levels is neglectable. Based on this, we perform the DGE analysis with univariate Poisson regression models for all genes. Under the target FDR level 0.10 with BH correction, 2085 genes are identified as differentially expressed genes between heterozygous OPCs (from Scn8a+/− mice) and wild-type OPCs (from Scn8a+/+ mice), which are shown in Fig. 5d and detailed in Table S3, available as supplementary data at *Bioinformatics* online. These identified differentially expressed genes can be either direct effect genes, indirect effect genes or passenger effects.

To identify direct effect genes, we first perform the lasso regression and narrow down to 375 genes (shown in in Fig. 5e) that are likely to have direct effects. However, we do not have rigorous guarantee on the proportion of false discoveries among these genes. Thus, we apply knockoff-based CDGE analysis on DGE Z-scores and expression level correlation matrix Σ of all genes. Here, Σ is estimated under a factor model (Leek and Storey 2007, Fan *et al.* 2020) with the estimated number of factors $\hat{r}=27$ using all cells. To account for the randomness of knockoff generation, we perform 100 independent runs of the knockoff-based CDGE analysis and summarize the 100 obtained selection sets under the target FDR level α=0.10 via the inclusion-rate based approach [Algorithm 2 of Dai *et al.* (2023)]. Figure 5f highlights all 167 CDGE-identified genes (under the target FDR level α=0.10) in the volcano plot, whose details are provided in Table S3, available as supplementary data at *Bioinformatics* online. As shown by the Venn diagram in Fig. 5c, all CDGE-identified genes are selected by the lasso regression, implying that the proportion of direct effect genes in the remaining 208 genes (identified by lasso but not CDGE) should be under 90%. Accounting for correlations of expression levels in our analysis, we eliminate genes’ marginal associations with epilepsy that are brought by their correlations to direct effect genes. As a result, we identify 167 direct effect genes (32 of which are not identified in DGE analysis) while 1950 significantly differentially expressed genes are not identified in the knockoff-based CDGE analysis. This suggests that more than 90% of differentially expressed genes have either indirect effects or passenger effects mediated by CDGE-identified genes. To validate this statement, we adjust for 167 CDGE-identified genes and perform DGE analysis on the remaining genes, with detailed procedures in Section 2.4.2. As shown in Fig. 5g, marginal associations between 91.75% of differentially expressed genes and epilepsy are decreased by adjusting for the 167 CDGE-identified genes. This validates our previous statement that effects of more than 90% differentially expressed genes are mediated.

As CDGE analysis targets direct effect genes, it is expected that CDGE-identified genes are more informative with respect to biological processes of OPCs, including proliferation, differentiation, and myelination. To verify the biological relevance of CDGE-identified genes, we perform an enrichment analysis. Here, we use the gene set library “GO_Biological_Process_2025” (Ashburner *et al.* 2000, Aleksander *et al.* 2025) in the “Enrichr” database (Chen *et al.* 2013, Kuleshov *et al.* 2016, Xie *et al.* 2021) for analysis. Detailed information of these pathways with respect to biological processes of OPCs is provided in Table S4, available as supplementary data at *Bioinformatics* online. Specifically, as the gene set library “GO_Biological_Process_2025” corresponds to human genes while all our CDGE-identified genes are mouse genes, the enrichment analysis is performed on their human orthologs because GO biological processes are highly conserved across mammals. Figure 5h–i present the enrichment of human orthologs of CDGE-identified genes over pathways of neuron proliferation, neuron differentiation, myelination and oligodendrocyte development, in comparison against all genes and DGE-identified genes respectively. In general, human orthologs of CDGE-identified genes are enriched in almost all relevant pathways, especially in some differentiation pathways and all proliferation pathways, no matter compared against all genes or DGE-identified genes. Although not very large, log⁡(OR) of myelination and oligodendrocyte development are positive with values around one when comparing human orthologs of CDGE-identified genes against DGE-identified ones. For example, our CDGE identifies fundamental genes with human orthologs for myelin sheaths formation and maintenance in deep white matter (MBP and PLP1) and early-stage differentiation of OPCs (NCAM1) (Harlow *et al.* 2014, Tisoncik-Go *et al.* 2024). All these results are consistent to the existing discovery that epilepsy causes hypermyelination of seizure circuits related to neuron differentiation, myelination, and oligodendrocyte development. In contrast, differentially expressed genes’ human orthologs SLC17A7 and CPLX1, which relate to oligodendrocyte lineage and synaptic signaling, are not identified in CDGE analysis, suggesting these genes are not likely to have direct effects to epilepsy. Such a case is true even for several down-regulated genes with the smallest *P* value in the DGE test, which means that the most significant differentially expressed genes may fail to capture direct effects. Although we can expect there remain several direct effect genes unidentified (within those identified by lasso but not CDGE), effects of these false negatives should be neglectable as shown in Fig. 5j, where the differentially expressed genes not identified by CDGE have lower enrichment level than CDGE-identified genes in most relevant pathways.

### 3.4. Applications to genome-scale perturb-seq experiments

In addition to RNA sequencing datasets that investigate gene-disease relations, we apply the knockoff-based CDGE analysis to the genome-wide perturb-seq dataset collected by Replogle *et al.* (2022) that consists of 2273 gene perturbation experiments for gene–gene connection study. To investigate functions of poorly characterized genes and uncover new regulators of various molecules activities (e.g. ribosome biogenesis, transcription, and mitochondrial respiration), Replogle *et al.* (2022) collected expression levels of human genes within CRISPR interfered cells in chronic myeloid leukemia and retinal pigment epithelial cell lines. Specifically, for each gene of interest, they perform loss-of-function perturbation on it in several cells (5 ∼ 1996) and compare the expression levels of all genes in these cells to control cells which are not perturbated. In our analysis, we use the pseudo-bulk dataset that records pseudo-bulk gene expressions in cells sampled in the sixth day after transduction. DGE Z-scores of 8563 genes are provided in Replogle *et al.* (2022) by comparing the perturbed cells in each experiment to the 10 691 control cells via the t-test. We filter out experiments with potentially inflated Z-scores, defined as those with median of $Z_{j}^{2}$ larger than 70%-quantile of $\chi_{1}^{2}$ distribution. This results in 1284 “P1P2” experiments with perturbation targeted at the consensus transcription start site, which would simultaneously perturb the primary and the secondary transcription start sites that are less than 1 kb apart (Horlbeck *et al.* 2016). Brief visualization of these Z-scores is provided in Fig. 6a. Among the experiments, 123 043 gene–gene connections (95.83 per experiments) are identified by DGE analysis under the target FDR level 0.05 with BH correction.

**Figure 6:**
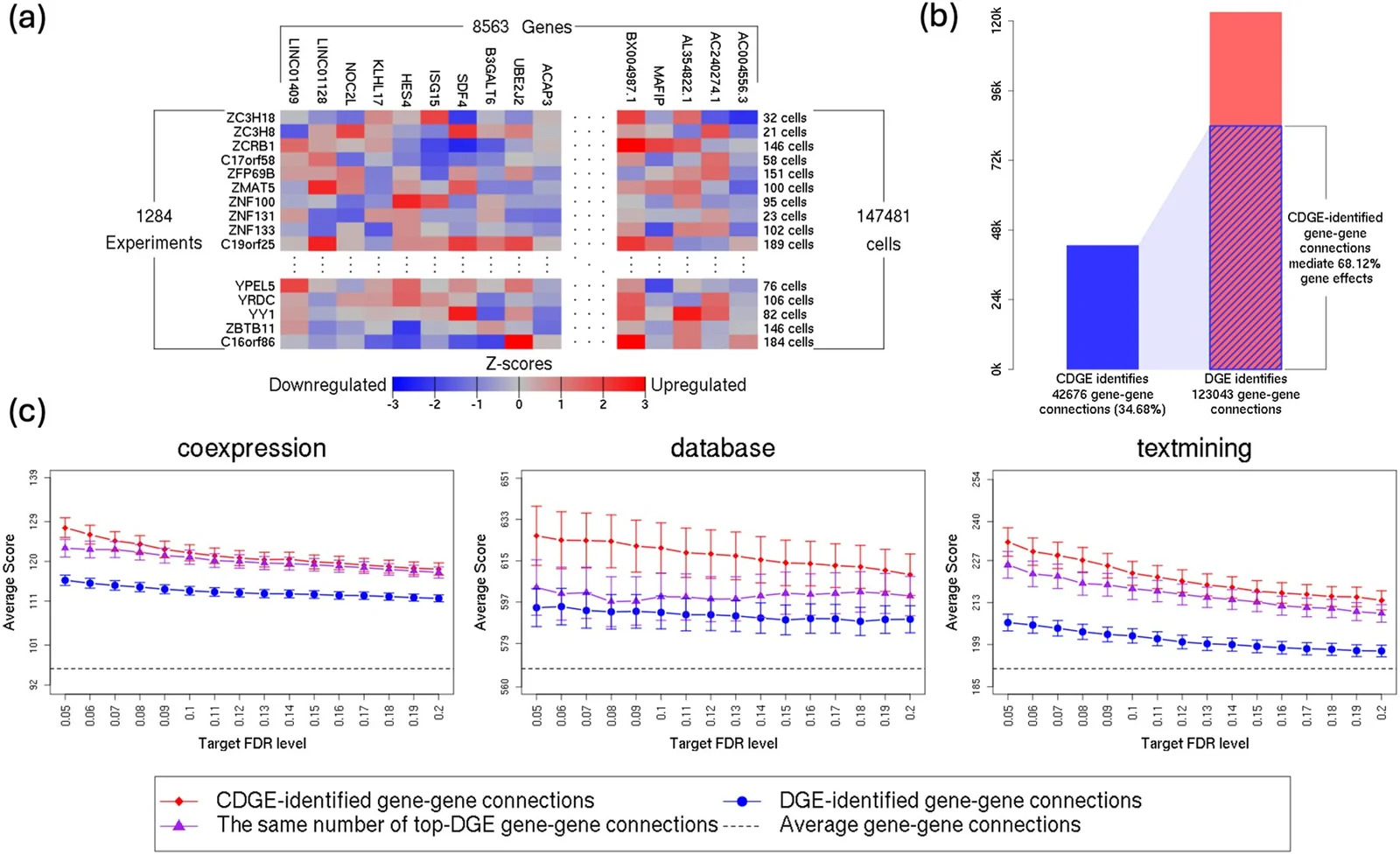
(a) Visualization of differential expression Z-scores over 1284 “P1P2” experiments in comparison to control cells. (b) Comparison of the number of CDGE-identified gene–gene connections, the number of DGE-identified gene–gene connections and the number of gene–gene connections whose effect is mediated by CDGE-identified gene–gene connections. (c) Confidence scores of protein–protein interactions that correspond to gene–gene connections discovered by knockoff-based CDGE analysis and DGE analysis under different target FDR levels over different evidence channels of the STRING network.

For each experiment, we apply the knockoff-based CDGE analysis on the corresponding Z-scores and expression level correlation matrix Σ of all genes to identify gene–gene connections of direct effects. Here, Σ is estimated under a factor model (Leek and Storey 2007, Fan *et al.* 2020) with the estimated number of factors $\hat{r}=77$ using all control cells. We use the same Σ  throughout all experiments. Under the target FDR level α=0.05, the number of identified gene–gene connections is 42 676 (33.24 per experiments). Details of all these CDGE-identified gene–gene connections are provided in Table S5, available as supplementary data at *Bioinformatics* online. Such an around 65% decrease is consistent to both the simulation study and analysis of the psoriasis dataset and epilepsy dataset, where most of differentially expressed genes are only of indirect effects or of passenger effects. To validate whether these CDGE-identified gene–gene connections capture direct effects between genes and mediate other gene–gene interactions, we adjust for CDGE-identified genes in each experiment and perform DGE analysis on the remaining genes, with detailed procedures in Section 2.4.2. Figure 6b shows that these CDGE-identified gene–gene connections only take 34.73% of DGE-identified gene–gene connections in number but explain around 70%. This suggests that the knockoff-based CDGE analysis can precisely identify gene–gene expressions that capture direct effects and mediate other gene–gene interactions.

We further validate whether the gene–gene connections discovered by the knockoff-based CDGE analysis are more biologically informative. To do so, we perform a series of enrichment analysis with respect to existing biological knowledges. First, we perform an enrichment analysis using the STRING protein–protein interaction (PPI) network (https://string-db.org/, version 12.0; Von Mering *et al.* 2005, Szklarczyk *et al.* 2023) to investigate whether CDGE-identified gene–gene connections are more informative to protein–protein interactions. In the STRING network, 6 857 702 functional associations among 19 699 proteins are annotated in human cells with confidence scores over various evidence channels, including:

1. The *coexpression* channel recoding the coexpression network with evidence from RNA expression arrays and RNA-Seq datasets processed by the GEO database (Barrett *et al.* 2013), scRNA-seq data in the Human Protein Atlas (Sjöstedt *et al.* 2020), co-regulation network of the ProteomeHD database (Kustatscher *et al.* 2019), and proteomics datasets of the PRIDE database (Perez-Riverol *et al.* 2022).
2. Two channels embedding existing knowledge of protein–protein associations, where

- The *database* channel records well-established knowledge of protein complexes, pathways and functional connections from KEGG (Kanehisa *et al.* 2004), Reactome (Gillespie *et al.* 2022), MetaCyc (Caspi et al. 2020), EBI Complex Portal (Meldal et al. 2022), and Gene Ontology Complexes (Ashburner *et al.* 2000, Aleksander *et al.* 2025).
- The *textmining* channel captures the cooccurrence of the gene/protein names in literature collected by the PMC Open Access Subset (up to April 2022), PubMed abstracts (up to August 2022), OMIM (Amberger *et al.* 2019), and SGD (Cherry *et al.* 2012).

In the enrichment analysis, we compute the average scores over all channels for protein–protein interactions

- corresponding to CDGE-identified gene–gene connections over α=0.05, 0.06,…,0.20;
- corresponding to the same number of top-DGE gene–gene connections; and
- corresponding to DGE-identified gene–gene connections under FDR levels 0.05, 0.06,…,0.20.

Average scores of all STRING-recorded interactions over all channels are also computed for reference. Figure 6c presents average scores of protein–protein interactions corresponding to different subsets of gene–gene connections in channels *coexpression*, *database*, and *textmining*. CDGE-identified gene–gene connections have higher confidence scores than DGE-identified connections under the same target FDR level. This suggests that a lot of biological uninformative gene–gene connections are identified by the DGE analysis, especially in terms of expression patterns and functional connections (in biological pathways and protein complexes). Even if we focus on the most significant DGE-identified connections of the same number as CDGE-identified connections, scores of CDGE-identified connections still dominate in the channels *database* and *textmining*.

We also consider biological pathway libraries under the Molecular Signatures Database (MSigDB; Subramanian *et al.* 2005, Liberzon *et al.* 2011, 2015) in our enrichment analysis. Specifically, we compare the enrichment of CDGE-identified gene–gene connections to DGE-identified ones in both overlapping and average PPI scores within:

1. All pathways from the Hallmark gene sets (Liberzon *et al.* 2015);
2. All pathways of malignant cells mined from the Curated Cancer Cell Atlas (3CA) metaprograms (Gavish *et al.* 2023);
3. All pathways of cancer modules identified in Segal *et al.* (2004) that changed significantly in various cancer conditions;
4. All pathways in the Biological Process subcollection derived from the Gene Ontology (GO) Consortium (Ashburner *et al.* 2000, Aleksander *et al.* 2025);
5. All pathways in the Cellular Component subcollection derived from the GO Consortium;
6. All pathways in the Molecular Function Component subcollection derived from the GO Consortium; and
7. All pathways in the Human Phenotype Ontology (Köhler *et al.* 2021).

The number of pathways in each library is presented in the second column of Table 1. In each pathway, we compute the log odd ratio of overlapping for both CDGE-identified gene–gene connections and DGE-identified ones. To compare the degree of enrichment, we perform the one-sided Wilcoxon test (against the null that “CDGE-identified gene–gene connections are at most as enriched as DGE-identified ones”) on log(OR)’s of all pathways in each library and present all the p-values in the third column of Table 1. We can find that CDGE-identified gene–gene connections are significantly more enriched than DGE-identified ones in pathways of all libraries. In addition, we query PPI scores of these overlapping gene–gene connections in the channels *coexpression*, *database*, and *textmining* of STRING, compute the combined score for each gene–gene connection and present one-sided paired Wilcoxon test p-values within different libraries in the fourth column of Table 1. We find that CDGE-identified gene–gene connections have significantly higher PPI scores than DGE-identified ones in pathways of all libraries except the Hallmark gene sets and cancer modules. In summary, gene–gene connections identified by CDGE are more biologically informative than DGE.

**Table 1:** p -values of one-sided paired Wilcoxon tests that compare the degree of enrichment of CDGE-identified gene–gene connections with DGE-identified ones in terms of overlapping log (OR)’s or PPI scores (significant results are highlighted in bold).

Library	No. of pathways	Overlapping log (OR)’s	PPI scores
Hallmark	50	$7.73\times 10^{-11}$	$9.14\times 10^{-2}$
Malignant cells pathways in 3CA gene sets	40	$1.91\times 10^{-8}$	$2.60\times 10^{-2}$
Cancer modules	431	$<2.20\times 10^{-16}$	$5.86\times 10^{-2}$
GO biological process	7583	$<2.20\times 10^{-16}$	$<2.20\times 10^{-16}$
GO cellular component	1042	$<2.20\times 10^{-16}$	$<2.20\times 10^{-16}$
GO molecular function	1855	$<2.20\times 10^{-16}$	$2.38\times 10^{-7}$
Human phenotype ontology	5748	$<2.20\times 10^{-16}$	$3.46\times 10^{-2}$

## 4. Discussions

In this article, we demonstrate the limitation of differential gene expression (DGE) analysis in learning biological effects of genes. Specifically, we show that differentially expressed genes between cell of different conditions can have either direct effects, indirect effects or passenger effects. This suggests DGE’s lack of ability to distinguish direct effect genes from other differentially expressed genes. To address this issue, we propose the conditional differential gene expression (CDGE) analysis, which infers conditional associations between genes and the trait of interests or among genes with expression level correlations into account. Using a knockoff-based approach, we show in extensive simulations that CDGE analysis can identify direct effect genes with guaranteed false discovery rate control while DGE analysis cannot. Analyses of observational RNA sequencing datasets of psoriasis and epilepsy and the experimental perturb-seq dataset validate our statement, as CDGE-identified genes (or gene–gene connections) does mediate effects of other genes (or gene–gene connections) and are more biologically informative.

These findings have direct implications for how RNA sequencing results are used in practice. If most differentially expressed genes are passengers rather than drivers, standard practices are systematically biased toward indirect signals: validating top DEGs experimentally, ranking drug targets by *P* values, and interpreting enrichment results across all DEGs and give disproportionate weight to mediated rather than direct effects. The same logic applies to gene–gene regulatory inference from perturb-seq, where the majority of measured downstream changes reflect propagation through the co-expression network rather than direct regulatory effects. CDGE provides a path to redirect follow-up effort toward genes more likely to be biologically causal, with corresponding gains in efficiency for functional validation and therapeutic development. Beyond the specific method, the direct/indirect/passenger/no-effect taxonomy we introduce gives the field a vocabulary for reasoning about what differential expression results mean, regardless of which conditional inference procedure is used to operationalize.

Several limitations of the present analysis are worth noting. First, our simulation evaluates CDGE under a single hierarchical generative model, and we have not systematically tested its robustness to model misspecification (for example, when expression follows a negative binomial or zero-inflated distribution), or extreme low n/p regimes that arise in some single-cell applications. Practitioners applying CDGE in such regimes should view our FDR guarantees as a target rather than an assured property and verify behavior in their setting where possible. Second, the mediation results we report (particularly the strong claim that adjusting for CDGE-identified genes eliminates the marginal significance of the remaining differentially expressed genes) are based on a single conditioning analysis and have not been benchmarked against random covariate-set baselines; they should be read as suggestive of mediation rather than as a definitive causal decomposition.

Another possible direction lies on the identification of the whole biological pathways by investigating mediated effects. As in Figs 1 and 3a, direct effect genes only include the most downstream of biological pathways, while existing methods cannot rigorously identify those indirect effect genes on the upstream. The key obstacle is that both the indirect effect genes and passenger genes possess the same statistical property under either DGE or CDGE framework: they are both marginally associated with but conditionally independent to the response of interest. As a result, for those differentially expressed genes not selected by CDGE analysis, further analysis is needed to understand their roles in the disease. One possible solution might be the development of sequential CDGE analysis with statistical guarantee for biological pathways detection, which might help to detail the biological mechanisms of gene effects mediation and identify drug targets for medical practice with both effectiveness and efficiency.

Finally, the conditional association between genes and the trait detected by CDGE analysis cannot be simply interpreted as causality, unless further assumptions are imposed. Following the potential outcomes framework (Rubin 1974, 2005; Imbens and Rubin 2015), we can let $Y_{x}$ denote the potential binary outcome of a cell that would have been observed if the gene expression levels are x. Under the assumptions that

1. **(Absence of unmeasured confoundings)**  $Y_{x}⊥X$;
2. (**Positivity**) P(X=x)>0 for all possible values x of X; and
3. (**Consistency**) the observed $Y=Y_{x}$ if X=x,

the conditional association between gene $X_{j}$ and Y can be translated to causal effects that


$$Y_{\left(x_{j},x_{-j}\right)}\neq Y_{\left(x_{j}^{*},x_{-j}\right)},\;\mathrm{for}\;\mathrm{some}\;x_{-j}\;\mathrm{and}\;x_{j}\neq x_{j}^{*}.$$

We emphasize that these assumptions, in particular the absence of unmeasured confounding, are unlikely to hold exactly in observational transcriptomics. Unmeasured cell-state variables, technical batch factors, and shared upstream regulators can all violate this assumption, and positivity is similarly difficult to verify when expression levels are continuous and high-dimensional. We therefore view the causal interpretation as an idealized benchmark rather than a routine claim of CDGE outputs. In practice, the conditional independence tested by CDGE should be understood as identifying direct statistical effects under the assumed correlation structure, with perturb-seq experiments providing the closest realistic approximation to the causal interpretation because the perturbation itself is a designed intervention. For purely observational RNA sequencing applications, conditional effects are best read as more biologically informative than marginal ones, without claiming full causal identification. This leaves an open question of how to eliminate confounding effects when using CDGE analysis to investigate biological causality in practice. In RNA sequencing datasets, there usually exist two types of confoundings that blur the identification and interpretation of direct effects, including technical confoundings caused by sequencing depth variation (Sarkar and Stephens 2021, Su *et al.* 2023) and biological confoundings caused by unmeasured biological processes. In addition, measurement errors in counting data make inference of direct effects much challenging, because errors not only attenuate direct effects but also mask the conditional independence between the trait of interest and the genes without direct effects (Wang *et al.* 2018, Zhang *et al.* 2020). Although out of the scope of this article, it is still desirable to extend CDGE analysis to eliminate unmeasured confounding in distilling causal effects.

## Supplementary Material

btag784_Supplementary_Data

## Data Availability

To facilitate public access to knockoff-based CDGE analysis, we have developed the R package “KnockoffCDGE” as an integrated implementation, which is freely available at https://github.com/GuJQ5/KnockoffCDGE (DOI: 10.5281/zenodo.23241517).
