# Supplementary material for: Distilling direct effects via conditional differential gene expression analysis
Source: Bioinformatics. 2026 Oct 20;42(11):btag784. doi: 10.1093/bioinformatics/btag784 (PMC13756079; doi:10.1093/bioinformatics/btag784)
Supplement: Supplementary file 1 [file btag784_supplementary_data.zip › Supplementary Figures (Oct 8 2026).docx]

**Supplementary Figures of “Distilling Direct Effects via Conditional Differential Gene Expression Analysis”**

**Figure S1**. **(a)** Empirical FDR of the single-knockoff implementation ($M=1$) and the multiple-knockoffs implementations with $M=2,3,4,5,10,50,100$ in identifying direct effect genes over all target FDR levels $\alpha\in\left[ 0,0.2 \right]$. **(b)** Empirical power of the single-knockoff implementation ($M=1$) and the multiple-knockoffs implementations with $M=2,3,4,5,10,50,100$in identifying direct effect genes over all target FDR levels $\alpha\in\left[ 0,0.2 \right]$. **(c)** Average stability index of selection sets (under the target FDR level $\alpha=0.1$) across independent runs of different implements on the same dataset. The average value is computed over 1000 datasets. **(d)** Average computational time of different implements of the knockoff-CDGE analysis over 1000 datasets. (CPU: AMD EPYC 7502 32-Core Processor 2.5GHz; RAM: 16GB)

**Figure S2**. **(a)** Barplot of the estimated number of factors $\hat{r}$ using the GR criterion of Ahn and Horenstein (2013). **(b)** The empirical FDR of the knockoff-based CDGE analysis with estimated $\hat{r}$ and the fixed $r=1,\ldots,10$ under the target FDR level $\alpha=0.10$. **(c)** The empirical FDR of the knockoff-based CDGE analysis with estimated $\hat{r}$ and the fixed $r=1,\ldots,10$ under the target FDR level $\alpha=0.20$. **(d)** The empirical power of the knockoff-based CDGE analysis with estimated $\hat{r}$ and the fixed $r=1,\ldots,10$ under the target FDR level $\alpha=0.10$. **(e)** The empirical power of the knockoff-based CDGE analysis with estimated $\hat{r}$ and the fixed $r=1,\ldots,10$ under the target FDR level $\alpha=0.20$.

**Figure S3**. **(a)** Empirical FDR of the knockoff-based CDGE analysis under different sample sizes ($n$) and numbers of genes ($p$) over all target FDR levels $\alpha\in\left[ 0,0.2 \right]$. **(b)** Empirical power of the knockoff-based CDGE analysis under different sample sizes ($n$) and numbers of genes ($p$)over all target FDR levels $\alpha\in\left[ 0,0.2 \right]$. (*When* $p=1000$*, the data is generated under the same mechanism described in “Experiments” section except that the number of direct effect genes, indirect effect genes, passenger genes and no effect genes are doubled in each layer.*)

**Figure S4**. **(a)** Empirical FDR of the knockoff-based CDGE analysis under different numbers of direct effect genes ($k$) over all target FDR levels $\alpha\in\left[ 0,0.2 \right]$. **(b)** Empirical power of the knockoff-based CDGE analysis under different numbers of direct effect genes ($k$) over all target FDR levels $\alpha\in\left[ 0,0.2 \right]$. (*For each candidate value of the number of direct effect genes, we adjust the number of passenger genes as* $20-0.2\cdot k$ *correspondingly in each layer, such that the total number of genes remains as* $p=500$*.*)

**Figure S5**. **(a)** Empirical FDR of the knockoff-based CDGE analysis under different signal strengths ($a$) and the target FDR level $\alpha=0.2$. **(b)** Empirical power of the knockoff-based CDGE analysis under different signal strengths ($a$) and the target FDR level $\alpha=0.2$. (*For each candidate value of signal strength* $a$*, we simulate 1000 datasets with* $\beta_{451},\ldots,\beta_{500}\sim N\left( 0,0.004\cdot a \right)$*, such that* $a=5$ *corresponds to the setting described in “Experiments” section.*)

**Figure S6**. **(a)** Empirical FDR of the knockoff-based CDGE analysis under different $\lambda_{E}$’s and the target FDR level $\alpha=0.2$. **(b)** Empirical power of the knockoff-based CDGE analysis under different $\lambda_{E}$’s and the target FDR level $\alpha=0.2$. (*For each candidate value of* $\lambda_{E}$*, we simulate 1000 datasets with* $E_{\mathrm{direct}}$ *being a 50-dimensional vector of i.i.d.* $Pois\left( \lambda_{E} \right)$ *random variables, such that* $\lambda_{E}=2/2$ *corresponds to the setting described in “Experiments” section. Here, the smaller* $\lambda_{E}$ *is, the stronger collinearity exists among direct effect genes.*)
